# Cholesterol Targeted Catalytic Hydrogel Fueled by Tumor Debris can Enhance Microwave Ablation Therapy and Anti‐Tumor Immune Response

**DOI:** 10.1002/advs.202406975

**Published:** 2024-12-12

**Authors:** Lin Shen, Zhijuan Yang, Yi Zhong, Yanran Bi, Junchao Yu, Qinwei Lu, Yanping Su, Xiaoxiao Chen, Zhongwei Zhao, Gaofeng Shu, Minjiang Chen, Liang Cheng, Liangzhu Feng, Chenying Lu, Zhuang Liu, Jiansong Ji

**Affiliations:** ^1^ Zhejiang Key Laboratory of Imaging and Interventional Medicine, Zhejiang Engineering Research Center of Interventional Medicine Engineering and Biotechnology The Fifth Affiliated Hospital of Wenzhou Medical University Lishui 323000 P. R. China; ^2^ Clinical College of The Affiliated Central Hospital School of Medicine, Lishui University Lishui 323000 P. R. China; ^3^ Institute of Functional Nano & Soft Materials (FUNSOM), Jiangsu Key Laboratory for Carbon‐Based Functional Materials & Devices Soochow University Suzhou Jiangsu 215123 P. R. China

**Keywords:** cholesterol‐targeted catalytic hydrogel, ferroptosis, ICIs, MWA, synergistic therapy

## Abstract

The immunosuppressive residual tumor microenvironment (IRTM) is a key factor in the high recurrence and metastasis rates of hepatocellular carcinoma (HCC) after microwave ablation (MWA). Cholesterol‐rich tumor fragments significantly contribute to IRTM deterioration. This study developed a cholesterol‐targeted catalytic hydrogel, DA‐COD‐OD‐HCS, to enhance the synergy between MWA and immune checkpoint inhibitors (ICIs) for HCC treatment. Cholesterol oxidase (COD), modified with dimethyl maleic anhydride (DA) for release in acidic IRTM, is used to degrade cholesterol. Oxydextran (OD) and hemin‐chitosan (HCS) formed a dual network gel, ensuring long‐term fixation of COD and hemin in the IRTM post‐MWA. In both in vitro and in vivo HCC models, DA‐COD‐OD‐HCS effectively released COD, degraded cholesterol, and induced tumor cell ferroptosis, enhancing the antitumor immune response. Combined with anti‐PD‐L1 immunotherapy, this strategy inhibited primary tumor growth and distant metastases, without side effects on adjacent tissues. This work highlights that cholesterol‐targeting catalytic hydrogels fueled by tumor debris can significantly improve the efficacy of MWA and ICIs, offering a novel therapeutic approach for HCC.

## Graphical abstract

This research introduces a cholesterol‐targeted catalytic hydrogel (DA‐COD‐OD‐HCS) designed to enhance microwave ablation (MWA) therapy and antitumor immunity in hepatocellular carcinoma (HCC). Using cholesterol from tumor debris as fuel, the hydrogel facilitates ferroptosis, reshapes the tumor microenvironment, and promotes the effectiveness of immune checkpoint inhibitors, offering a promising therapeutic approach to reduce HCC recurrence and metastasis.

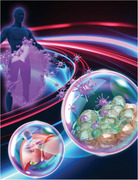

## 1 Introduction

Hepatocellular carcinoma (HCC) is a highly prevalent and lethal digestive system tumor, ranking 7th in incidence and 2nd in mortality among cancers globally, with ≈910 000 new cases annually.^[^ ^1^ ^]^ Over 830 000 HCC‐related deaths occur each year, with nearly half in China, posing a significant health threat.^[^ ^2^ ^]^ While surgical resection remains the ideal treatment, achieving a cure, less than 20% of Chinese patients are eligible due to late‐stage diagnosis and complications like hepatitis B and liver cirrhosis.^[^ ^3^ ^]^ Microwave ablation (MWA) is a minimally invasive interventional technique for tumor ablation using heat from the oscillation of polar molecules under microwave irradiation.^[^ ^4^ ^]^ MWA offers less trauma, high efficiency, and fewer complications, making it a key treatment for unresectable HCC and other solid tumors, such as lung and colorectal cancer metastases.^[^ ^4^, ^5^ ^]^ Despite these benefits, HCC recurrence and metastasis rates post‐MWA remain high, particularly for lesions over 3 cm in diameter, with a 60% chance of distant metastasis within three years.^[^ ^6^ ^]^ The irregular shape of tumors often necessitates a large ablation zone, leading to significant thermal damage to surrounding tissues and frequent side effects.^[^ ^4^ ^]^ Therefore, improving the anticancer efficacy of MWA is crucial to addressing these challenges and achieving safer, more effective cancer treatments.

Activating the antitumor immune response post‐MWA is a promising strategy for preventing tumor recurrence and metastasis.^[^ ^7^ ^]^ While local thermal ablation therapies like MWA generate tumor antigens and damage‐associated molecular patterns (DAMPs), the immune response they induce is often weak, limiting their efficacy in inhibiting tumor progression.^[^ ^4^, ^8^ ^]^ Although immune checkpoint inhibitors (ICIs) can enhance this response, the overall benefit for HCC patients is limited, with only 15∼20% responding effectively.^[^ ^9^ ^]^ Thus, developing adjuvant biomaterials tailored to the post‐MWA tumor microenvironment, which possess both microwave susceptibility and immunostimulatory properties, is crucial for enhancing the synergistic antitumor effects of MWA and ICIs. The immunosuppressive tumor microenvironment, driven by high cholesterol levels, significantly restricts the synergistic antitumor effects of MWA and ICIs.^[^ ^10^ ^]^ Cholesterol, a critical cell membrane component, is abundantly expressed in tumor tissues, supporting rapid tumor cell proliferation and migration.^[^ ^11^ ^]^ High cholesterol intake by CD8^+^ T cells caused endoplasmic reticulum (ER) stress and depletion, reducing their effectiveness against tumors.^[^ ^10a^ ^]^ Additionally, cholesterol recruits immune‐suppressive cells, aids tumor immune escape, and decreases C‐C chemokine receptor type 7 (CCR7) expression in dendritic cells, impairing their migration and antitumor response.^[^ ^10^, ^12^ ^]^ The release of cholesterol from tumor debris post‐MWA exacerbates immunosuppression, highlighting the need for innovative treatments targeting cholesterol metabolism to enhance cancer therapy outcomes.

To effectively reduce cholesterol levels and reshape the immunosuppressive tumor microenvironment after MWA, cholesterol oxidase (COD) was introduced to catalyze cholesterol degradation. COD, a flavoprotein oxidoreductase, converts cholesterol to 4‐cholestene‐3‐one and H_2_O_2_, effectively removing cholesterol from the tumor microenvironment.^[^ ^13^ ^]^ Notably, 4‐cholestene‐3‐one exhibits antitumor effects by promoting the release of high mobility group box 1 (HMGB1), blocking HIF‐1α nuclear translocation, and inhibiting MMP‐2 and MMP‐9 activation, thereby preventing tumor invasion and metastasis.^[^ ^14^ ^]^ Additionally, combining COD with hemin at the MWA ablation site induces a Fenton reaction with H_2_O_2_, leading to ferroptosis of residual cancer cells and enhancing antitumor immunity.^[^ ^15^ ^]^ Hemin, a metalloporphyrin compound with peroxidase‐like activity, converts H_2_O_2_ to •OH, inducing ferroptosis.^[^ ^15^, ^16^ ^]^ This process reshapes the tumor microenvironment by activating DC maturation, promoting T‐cell infiltration, and inducing macrophage M1 polarization, thus significantly enhancing the efficacy of immune checkpoint blockade therapy.^[^ ^17^ ^]^

**Figure 1:**
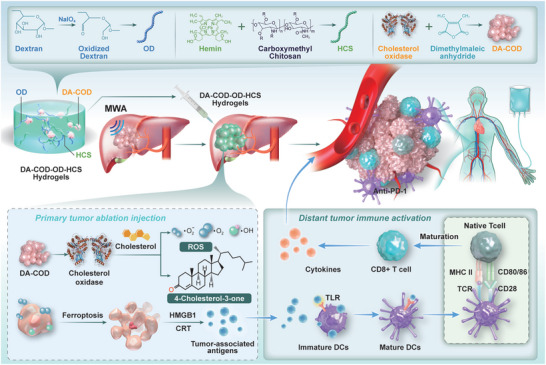
Schematic diagram illustrating how the cholesterol‐targeted catalytic hydrogel enhances MWA treatment and boosts antitumor immunity. Schematic diagram illustrating the preparation of a cholesterol‐targeted catalytic hydrogel for locoregional treatment of HCC. The novel injectable cholesterol‐catalyzed hydrogel system mainly includes DA‐COD, hemin, OD, and CS. First, dextran was oxidized to the OD by NaIO_4_. Second, HCSs were prepared by covalent coupling of hemin and CS through an amide reaction. Third, dimethyl maleic anhydride was used to modify the COD to construct DA‐COD. Ultimately, DA‐COD, OD, and HCS will form the cholesterol‐catalyzed hydrogel DA‐COD‐OD‐HCS in situ within the tumor, responding to the acidic residual microenvironment of cholesterol‐rich tumor debris after MWA. Schematic diagram of the synergistic effect of MWA and ICIs on cholesterol‐targeted hydrogels fueled by tumor debris. After MWA treatment, the HCC cells formed an acidic residual tumor microenvironment composed of cholesterol‐rich tumor debris. After local injection of the DA‐COD‐OD‐HCS hydrogel, DA‐COD releases COD under acidic conditions to degrade cholesterol in tumor fragments, eventually producing H_2_O_2_ and 4‐cholestene‐3‐one. On the basis of the antitumor effect of 4‐cholestene‐3‐one, hemin further reacts with H_2_O_2_ through the Fenton reaction to induce the ferroptosis of residual tumor cells and the production of tumor antigens or DAMPs. The release of tumor antigens, such as HMGB1 molecules, from ferroptotic cancer cells recruits immature DCs to the residual tumor site and primes specific antitumor immune responses characterized by increased infiltration of effector T cells and secretion of effector cytokines to further inhibit the growth of both residual tumors and metastatic (distant) tumors, especially in combination with anti‐PD‐L1 immunotherapy.

To enhance COD's response and release in the residual cancer microenvironment, we modified COD's amino group with dimethylmaleic anhydride (DA), ensuring its activity in the acidic tumor environment.^[^ ^18^ ^]^ Post‐MWA, DA‐COD was anchored in the residual tumor using our adhesive OD‐hemin‐CS (OD‐HCS). In the acidic, cholesterol‐rich residual tumor microenvironment, the cholesterol‐targeted catalytic hydrogel (DA‐COD‐OD‐HCS) efficiently released COD, degrading cholesterol and inducing tumor cell ferroptosis by improving the immunosuppressive environment. This enhanced the antitumor immune response, effectively boosting the synergistic anticancer effects of MWA and ICIs (**Figure** **1**).

## 2 Results

### 2.1 Design and Characterization of Cholesterol‐Targeted Catalytic Hydrogels

**Figure 2:**
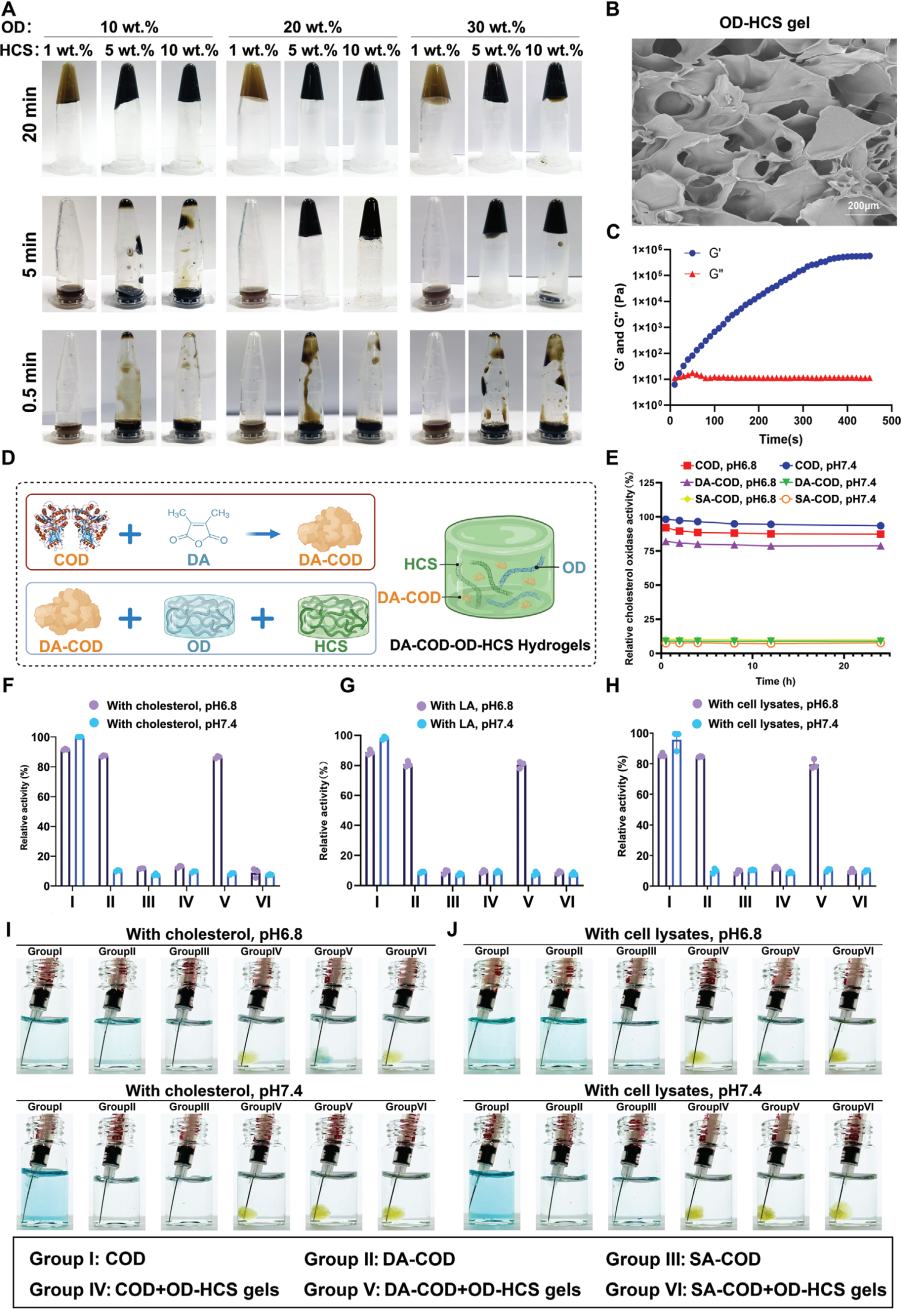
Design and characterization of the cholesterol‐fueled catalytic hydrogel. A) Images of hydrogels formed at 0.5, 5, and 20 min after mixing 10, 20, and 30 wt.% OD with 1, 5, and 10 wt.% HCS, respectively. B) SEM image of the OD‐HCS hydrogel. C) Storage modulus (G’) and loss modulus (G”) of OD‐HCS hydrogels according to time sweep measurements. D) Schematic illustration of the synthesis process of DA‐COD‐OD‐HCS hydrogels. E) The relative enzyme activities of COD, DA‐COD, and SA‐COD were assessed using an HRP‐ABTS assay at pH 7.4 and 6.8. F) An HRP‐ABTS assay was used to investigate the relative catalytic efficiency of each group on cholesterol at pH 7.4 and 6.8. G) HRP‐ABTS assay showing the relative catalytic efficiency of each group on linoleic acid (LA) at pH 7.4 and 6.8. H) HRP‐ABTS assay showing the relative catalytic efficiency of each group on cholesterol in cell debris at pH 7.4 and 6.8. I,J) Images of TMB containing various types of catalytic hydrogels or CODs after being immersed in cholesterol I) or cell lysate J) solutions at pH 7.4 and 6.8 for 5 min. Data are presented as the means ± standard errors of the means (SEMs), n = 3 biologically independent samples.

OD, CS, and hemin were used to enhance the retention and Fenton effect of COD to potentiate its antitumor effect in residual tumors after incomplete MWA (iMWA). Sodium periodate was used to oxidize dextran to obtain dextran aldehydes, which were then used to glycosylate the COD to improve the stability of the enzyme (Figure 1A). Hemin, an effective Fenton catalyst, was covalently coupled with CS by an amide formation reaction to obtain highly water‐soluble HCSs (Figure 1B). Then, the OD and HCSs were mixed to prepare the OD‐HCS hydrogel. FT‐IR spectrum analysis of commercial dextran and oxidized dextran revealed that OD formed an aldehyde group (1734 cm^−1^), which was attributed to COD glycosylation (Figure S1A, Supporting Information). After mixing OD (20 wt.%) and HCS (5 wt.%), the OD‐HCS hydrogel formed within 5 min, which is better for drug fixation to the residual tumor after iMWA (**Figure** **2**A). Although OD enhanced the stability of COD, OD inhibited the catalytic activity of natural enzymes to some extent, as 20 wt.% OD reduced the cholesterol‐catalytic activity of 5 U mL^−1^ COD to 9.6% (Figure S1B, Supporting Information). SEM revealed that the OD‐HCS hydrogels demonstrated a homogeneous porous network structure, whereas the OD and HCS hydrogels did not (Figure 2B; Figure S1C,D, Supporting Information). Rotational rheometry revealed that OD exhibited fluid behavior, with a loss modulus (G″) greater than the storage modulus (G″), and HCS exhibited elastic behavior, with a G″ greater than the G″ (Figure S1E,F, Supporting Information). As time progressed, the G′ of the OD‐HCS hydrogel progressively increased, whereas the G′ remained relatively unchanged, indicating a significant increase in the elastic behavior of the hydrogel following the formation of cross‐linked structures (Figure 2C).

DA‐COD was prepared by using 2.5 mg mL^−1^ DA and 5 mg mL^−1^ COD under alkaline conditions and then mixed with the OD‐HCS hydrogel to prepare a cholesterol‐targeted catalytic hydrogel (DA‐COD‐OD‐HCS, Figure 2D) to effectively protect the cholesterol‐catalytic activity of COD. Next, the catalytic activity of COD under physiological conditions was evaluated by measuring the H_2_O_2_ production of the mixture of COD and cholesterol at different pH values and temperatures. COD showed high catalytic activity at pH values ranging from 6∼8 and at reaction temperatures ranging from 37–45 °C, indicating its effective catalytic performance under physiological conditions (Figure S1G,H, Supporting Information).

To determine the acid response and release ability of DA‐COD, we investigated the cholesterol‐catalytic activity of DA‐COD at pH 6.8 and 7.4. Compared with COD, DA‐COD demonstrated ≈81.14% cholesterol‐catalytic activity at pH 6.8 but not at pH 7.4, indicating that DA‐COD responds well to the acidic tumor microenvironment and cholesterol‐catalytic ability (Figure 2E). Next, we investigated the acid response and cholesterol catalysis ability of DA‐COD mixed with OD‐HCS hydrogels. The DA‐COD‐OD‐HCS hydrogel demonstrated a better acid response and cholesterol‐catalytic activity at pH 6.8 than that observed in the succinic anhydride (SA) group with irreversible mercapto modification (Figure 2F,I). Interestingly, the DA‐COD‐OD‐HCS hydrogel demonstrated considerable catalytic ability to promote LA peroxidation at pH 6.8 (Figure 2G). When using tumor cell fragments as substrates, we demonstrated that the DA‐COD‐OD‐HCS hydrogels also showed catalytic activity and •OH production at pH 6.8 (Figure 2H,J). In summary, the DA‐COD‐OD‐HCS hydrogel more effectively promotes pH‐responsive lipid peroxidation than the COD/SA‐COD‐OD‐HCS hydrogel.

### 2.2 The Antitumor Effect of Cholesterol‐Catalzyed Coupling‐Induced Ferroptosis in Cancer Cells

**Figure 3:**
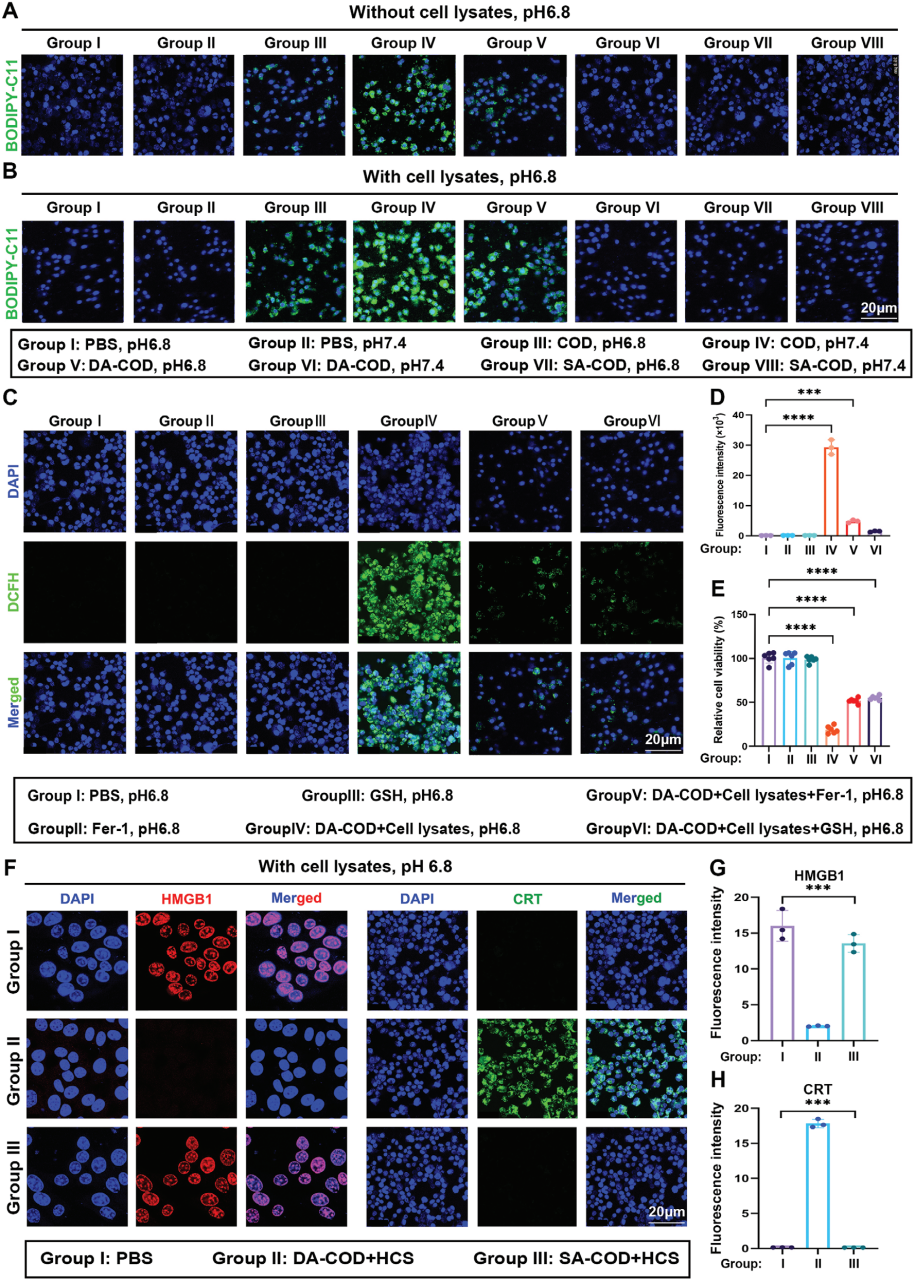
Cholesterol‐fueled catalytic hydrogels induce ferroptosis‐mediated cancer cell death. A,B) Confocal imaging of the intracellular lipid peroxidation of H22 cells subjected to different treatments, as indicated, in the absence A) or presence B) of cell lysates stained with the BODIPY‐C11 probe. C–E) Confocal imaging of intracellular lipid peroxidation C), fluorescence intensity D), and relative cell viability E) in H22 cells after various treatments as indicated. F–H) Confocal images of intracellular ferroptosis F) and fluorescence intensity G,H) in H22 cells after various treatments, as indicated. The data are presented as the means ± SEMs; *n* = 3 biologically independent samples. *p < 0.05, **p < 0.01, ***p < 0.001, ****p < 0.0001.

After applying COD and HCS to convert cholesterol into •OH through a highly efficient cascade reaction, we studied the antitumor effect of these compounds on tumor cell ferroptosis induced by tumor cell debris. Using commercial BODIPY‐C11 or DCFH‐DA as specific probes for intracellular lipid peroxidation, the effects of COD, DA‐COD, or SA‐COD fueled by cancer cell lysates on lipid peroxidation in cancer cells at pH 6.8 and 7.4 were carefully studied by confocal microscopy and flow cytometry analysis. The intracellular lipid peroxidation ability of DA‐COD (DA = 2 mg mL^−1^, COD = 5 U mL^−1^) in the presence of cell lysate was much greater than that in its absence (**Figure** **3**A,B). In addition, in H22 cells treated with cell lysis products (1 × 10^6^ dead cells), DA‐COD showed more effective intracellular lipid peroxidation than COD (COD = 5 U mL ^−1^) in an acidic environment at pH 6.8 (Figure S2 and S3, Supporting Information). Therefore, DA‐COD can effectively induce intracellular lipid peroxidation. Then, we investigated the rescuing effects of two effective ferroptosis inhibitors, ferrostatin 1 (Fer‐1) and glutathione (GSH), on cell death induced by DA‐COD plus lysate. Interestingly, through confocal microscopy observation and analysis using BODIPY‐C11 as a probe, it was found that the addition of both Fer‐1 and GSH could effectively suppress the production of intracellular lipid peroxides in H22 cells incubated with DA‐COD plus cell lysates (1 × 10^6^ cells) for 6 h in an acidic environment at pH 6.8. Consistently, Fer‐1 or GSH treatment could effectively reverse the cytotoxicity of DA‐COD plus cell lysate on H22 cells (Figure 3C–E), suggesting that DA‐COD and the tumor cell lysate are potent inducers of cell death via the ferroptosis pathway.

Confocal microscopy revealed that after H22 cells were treated with DA‐COD‐HCSs for 24 h in an acidic environment at pH 6.8, the release of HMGB1 from the cell nuclei significantly increased, and the expression of CRT increased, in sharp contrast to the findings observed after the other treatments, which had minimal influence on DAMPs (Figure 3F–H). Additionally, the excellent cytotoxicity of this combination treatment was confirmed in MCF‐7, 4T1, HeLa, and A549 cells (Figure S4, Supporting Information). The above results suggest that DA‐COD gradually releases COD in acidic tumor environments. DA‐COD‐HCS uses cell lysis products as the source of cholesterol, causing a chain reaction of lipid peroxidation, eventually leading to the death of cancer cells via the ferroptosis pathway (Figure S5, Supporting Information).

### 2.3 The In Situ Formation of a Cholesterol‐Catalyzed Hydrogel Combined with iMWA to Kill Tumors In Vivo

**Figure 4:**
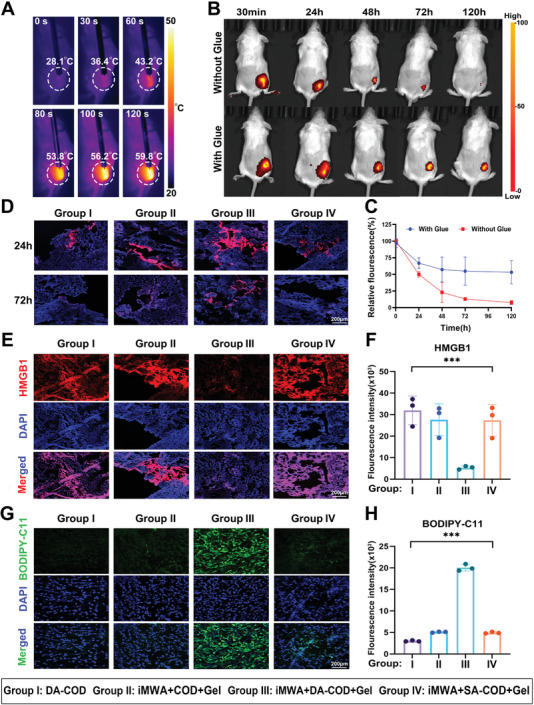
Intratumoral retention and ferroptosis‐inducing effects of the cholesterol‐fueled catalytic hydrogel after iMWA treatment in vivo. A) Infrared thermal images of H22 tumor‐bearing mice during iMWA treatment recorded at 0, 30, 60, 80, 100, and 120 s. B) In vivo fluorescence imaging of H22 tumor‐bearing mice intratumorally injected with Cy5.5‐labeled DA‐COD in the presence or absence of OD‐HCS adhesive glue at the indicated time points post iMWA treatment. Glue, OD‐HCSs. C) Semiquantitative analysis of the Cy5.5 fluorescence intensity in the tumor region based on the image in B). D) Confocal images of tumor slices collected from H22 tumor‐bearing mice intratumorally injected with various Cy5.5‐labeled agents as indicated at 24 and 72 h after different treatments. E,G) Confocal images of tumor slices collected from H22 tumor‐bearing mice after different treatments, as indicated, for 24 h and stained with HMGB1 (red, E) primary antibodies and corresponding Alexa‐488‐conjugated secondary antibodies and BODIPY‐C11 (green, G). F,H) Semiquantitative analysis of F) and H). The data are presented as the means ± SEMs; n = 3 biologically independent samples. *p < 0.05, **p < 0.01, ***p < 0.001, ****p < 0.0001.

To effectively ensure the antitumor ability of the cholesterol‐catalyzed hydrogel after iMWA, we carefully evaluated the retention and therapeutic effect of the DA‐COD‐OD‐HCS hydrogels in residual tumors after iMWA in vivo. H22 tumors (≈150 mm^3^) inoculated subcutaneously into BALB/c mice were heated using a commercial MWA system, and the temperature of the tumor area was controlled at ≈60 °C for 2 min by a thermal camera to establish a residual tumor model after iMWA (**Figure** **4**A). We used an IVIS in vivo fluorescence imaging system to monitor the fluorescence of covalently labeled Cy5.5 on COD to assess the intratumoral retention efficiency of the cholesterol‐catalyzed hydrogel. On the 5th day after the injection (p.i.), the DA‐COD‐OD‐HCS hydrogels retained ≈50% of the COD at the residual tumor site. In contrast, without using such a cholesterol‐catalyzed hydrogel, only ≈5% of the COD remained at the residual tumor site on the 5th day (Figure 4B,C). Furthermore, through laser confocal microscopy, we found that the cholesterol‐catalyzed hydrogel could still effectively promote the retention and lateral diffusion of Cy5.5‐labeled DA‐COD in residual tumors 72 h after iMWA, whereas Cy5.5 fluorescence was almost absent from tumor sections from mice injected with DA‐COD alone (Figure 4D). These results suggest that the DA‐COD‐OD‐HCS hydrogels can promote the long‐term retention and lateral spread of COD in residual iMWA‐treated tumors.

To determine the antitumor effect of the cholesterol‐catalyzed hydrogel combined with iMWA in vivo, we carefully evaluated the ability of the DA‐COD‐OD‐HCS hydrogels to induce intratumoral lipid peroxidation and immunogenic cell death (ICD) in cancer cells. H22 tumor‐bearing mice (≈150 mm^3^) were randomly divided into four groups and subjected to the following treatments: group I, DA‐COD; group II, iMWA + COD + OD‐HCS; group III, iMWA + DA‐COD + OD‐HCS; and group IV, iMWA + SA‐COD + OD‐HCS. First, an anti‐HMGB1 primary antibody was used for immunofluorescence staining to assess the efficacy of these treatments in inducing residual cancer cell ICD in vivo. Semiquantitative analysis revealed that the reduction in HMGB1 signal efficiency in tumor slices treated with iMWA combined with DA‐COD‐OD‐HCS (group III) was the greatest, at only 18.6%, compared with that in group I (Figure 4E,F). In addition, we found that the tumor slices from group III showed significantly stronger BODIPY‐C11 fluorescence than those from the other groups (Figure 4G,H). These results suggest that the cholesterol‐catalyzed hydrogel can induce effective ICD in cancer cells by initiating continuous lipid peroxidation after the local administration of iMWA.

**Figure 5:**
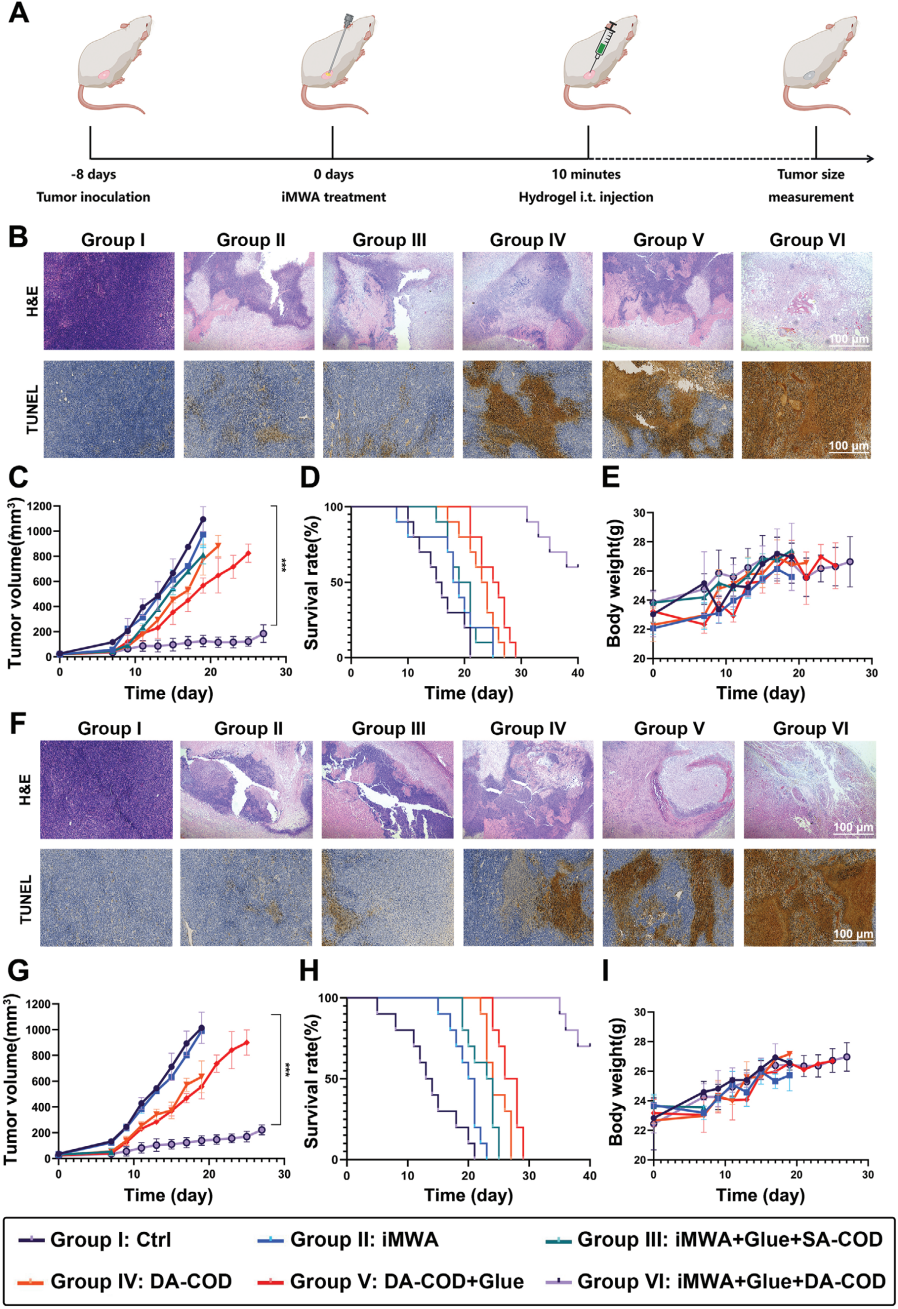
The ability of the cholesterol‐fueled catalytic hydrogel to enhance iMWA efficacy in a mouse H22 and 4T1 tumor model and an orthotopic liver cancer N1S1 rat tumor model. A) Schematic illustration of the in vivo therapeutic schedule for the mouse H22 or 4T1 tumor model. B,F) H&E and TUNEL staining of tumor slices collected from H22 B) or 4T1 F) tumor‐bearing mice after different treatments. C,G) Tumor growth curves of H22 C) and 4T1 G) tumor‐bearing mice after different treatments. D,H) Survival rates of H22 D) and 4T1 H) tumor‐bearing mice after different treatments. The mice were sacrificed when their tumor volume exceeded 1000 mm^3^. E,I) Mean body weight curves of H22 E) and 4T1 I) tumor‐bearing mice after different treatments, as indicated. Glue, OD‐HCS hydrogel. The data are presented as the means ± SEMs; n = 6 biologically independent samples. *p < 0.05, **p < 0.01, ***p < 0.001, ****p < 0.0001.

To evaluate the therapeutic effect of iMWA combined with DA‐COD‐OD‐HCS, thirty‐six mice bearing H22 tumors transfected with the NIR‐II fluorescent dye BBT‐2FT were randomly divided into six groups and subjected to the following treatments: group I, Ctrl; group II, iMWA; group III, iMWA + Glue + SA‐COD; group IV, DA‐COD; group V, DA‐COD + Glue; and group VI, iMWA + Glue + DA‐COD (Figure S6A, Supporting Information). Glue indicates the OD‐HCS hydrogel. By recording NIR‐II bioluminescence signals, it was semiquantitatively determined that ≈50% of the tumor masses were not destroyed after iMWA treatment, and the treatment applied to group VI (iMWA + Glue + DA‐COD) mice showed the most effective tumor inhibition effect (Figure S6B, Supporting Information). Furthermore, through H&E staining, we found that tumor sections collected from group VI mice showed the most severe histological damage. Microscopic examination with TUNEL staining further confirmed the superior therapeutic effect of this combination treatment in promoting tumor tissue apoptosis (**Figure** **5**A). The tumor growth rate of mice treated with the iMWA sequential cholesterol‐catalyzed hydrogel decreased significantly (Figure 5B). The median survival time of the mice treated with iMWA combined with cholesterol‐catalyzed hydrogel (group VI) was 45 days, significantly longer than that of the mice in group I (21 days) and the other treatment groups (Figure 5C). Additionally, this combination therapy did not affect the average body weight of any of the mice throughout the treatment, indicating that the cholesterol‐catalyzed hydrogel had good biocompatibility (Figure 5D).

To further clarify the synergistic effect of the cholesterol‐catalyzed hydrogel on iMWA in liver cancer, we combined iMWA plus cholesterol‐catalyzed hydrogel treatment in a 4T1 tumor model in mice (Figure S7A, Supporting Information). Thirty‐six 4T1 tumor‐bearing mice harboring the NIR‐II fluorescent dye BBT‐2FT were randomly divided into six groups and received the following treatments: group I, Ctrl; group II, iMWA; group III, iMWA + Glue + SA‐COD; group IV, DA‐COD; group V, DA‐COD + Glue; and group VI, iMWA + Glue + DA‐COD. Glue indicates the OD‐HCS hydrogel. By recording the NIR‐II bioluminescence signal, H&E staining, TUNEL staining, and tumor volume, we found that the combination of iMWA plus cholesterol‐catalyzed hydrogel had a greater tumor inhibitory effect than iMWA alone or intratumoral injection of DA‐COD, significantly prolonging the median survival time after iMWA (Figure 5E–G; Figure S7B, Supporting Information). Additionally, the body weights of all the mice showed no obvious variation during the therapeutic process (Figure 5H). The above results indicate that the intratumoral fixation of cholesterol‐catalyzed hydrogels is a promising strategy for improving the efficacy of conventional MWA for treating tumors.

**Figure 6:**
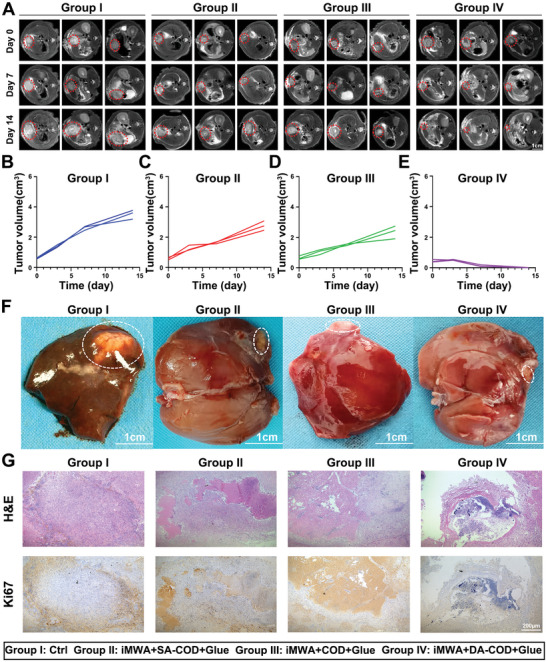
The cholesterol‐fueled catalytic hydrogel enhanced iMWA treatment in an orthotopic N1S1 tumor model. A) Representative 3.0‐T T2 MR image of N1S1‐bearing rats subjected to different treatments. B–E) Individual tumor growth curves (M: Ctrl, N: iMWA+SA‐COD+Glue, O: iMWA+COD+Glue, P: iMWA+DA‐COD+Glue) of different groups of N1S1‐bearing rats after various treatments as indicated. F) Representative optical images of N1S1 tumors collected from the aforementioned rats at 8 days after various treatments. G) H&E and Ki67 staining of tumor slices collected from N1S1 tumor‐bearing rats subjected to different treatments. The data are presented as the means ± SEMs; n = 6 biologically independent samples. *p < 0.05, **p < 0.01, ***p < 0.001, ****p < 0.0001.

Subsequently, the antitumor effect of iMWA combined with the cholesterol‐catalyzed hydrogel was further confirmed in a highly malignant N1S1 HCC rat tumor model. Twenty‐four SD rats bearing N1S1 liver tumors in situ were randomly divided into four groups and received the following treatments: group I, Ctrl; group II, iMWA + SA‐COD + Glue; group III, iMWA + COD + Glue; and group IV, iMWA + DA‐COD + Glue. The iMWA treatment was performed 8 days after the N1S1 orthotopic liver tumor model was established, and the tumor volume was ≈350 mm^3^. Hydrogels from each group were injected into the tumors on the day 0. On the day before treatment and the 7th and 14th days after different treatments, the rats were injected intraperitoneally with commercial gadolinium contrast agent and subjected to 3.0‐T MRI to record the tumor volumes. Group IV (iMWA + DA‐COD + Glue) demonstrated a strong antitumor effect on liver cancer, and 4 of the 5 rats were cured at 14 days after the corresponding treatment, while group II (iMWA + SA‐COD + Glue) and group III (iMWA + COD + Glue) treatments only partially inhibited HCC growth in situ (**Figure** **6**A–E). Finally, liver residual tumor observation, H&E staining, and Ki67 staining of the above 4 groups further confirmed that iMWA combined with cholesterol‐catalyzed hydrogel therapy could significantly inhibit the proliferation of HCC cells in situ by killing tumor tissue and had no obvious effects on the health status of the rats, such as body weight (Figure 6F,G; Figure S8, Supporting Information). In summary, our results demonstrate that MWA combined with a cholesterol‐catalyzed hydrogel can effectively inhibit tumor growth in situ without causing obvious side effects.

### 2.4 Combining In Situ Cholesterol‐Catalyzed Hydrogel Fixation and iMWA Treatment Boosted Antitumor Immunity

**Figure 7:**
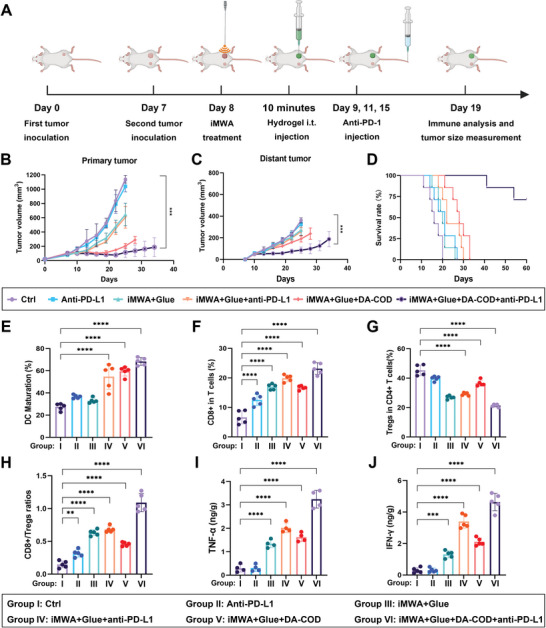
In vivo investigation into the antitumor efficacy and immune mechanisms of combined microwave ablation, cholesterol‐fueled catalytic hydrogels, and anti‐PD‐L1 immunotherapy. A) A schematic illustration of the inoculation of the bilateral tumor model for in vivo antitumor and immune mechanism studies. B–D) Tumor growth curves of primary B) and distant tumors C) and corresponding mobility‐free survival rates D) of mice with bilateral tumors after different treatments. The mice were considered dead when their tumor volume exceeded 1000 mm^3^. E) Flow cytometry statistical histogram showing the DC maturation status in the draining lymph nodes adjacent to the primary tumors after various treatments. F–H) Flow cytometry statistical histograms showing the frequencies of CD3^+^ CD8^+^ T cells F) and CD3^+^ CD4^+^ Foxp3^+^ Tregs G) and their ratios H) in distant tumors after various treatments. I,J) The TNF‐α I) and IFN‐γ J) secretion levels in distant tumors after various treatments. Glue, OD‐HCS hydrogels. The data are presented as the means ± SEMs; n = 5 biologically independent samples. *p < 0.05, **p < 0.01, ***p < 0.001, ****p < 0.0001.

To explore the ability of MWA combined with a cholesterol‐catalyzed hydrogel to enhance the antitumor immune response, we combined sequential iMWA and intratumoral cholesterol‐catalyzed hydrogel fixation with anti‐PD‐L1 immunotherapy (**Figure** **7**A). Thirty mice with two H22 tumors on each side were randomly divided into six groups (n = 5) and received the following treatments: group I, Ctrl; group II, anti‐PD‐L1; group III, iMWA + Glue; group IV, iMWA + Glue + anti‐PD‐L1; group V, iMWA + Glue + DA‐COD; and group VI, iMWA + Glue + DA‐COD + anti‐PD‐L1. Tumor size measurements revealed that after iMWA, intratumoral injection of a cholesterol‐catalyzed hydrogel combined with an anti‐PD‐L1 antibody more effectively suppressed the growth of both residual primary and distant tumors to a considerably greater extent than iMWA or anti‐PD‐L1 immunotherapy alone (Figure 7B,C). Four of the 5 mice treated with iMWA plus sequential cholesterol‐catalyzed hydrogel fixation and anti‐PD‐L1 injection (group VI) were cured, whereas 2 of the 5 mice treated with sequential iMWA and cholesterol‐catalyzed hydrogel (group V) were cured, and the cured mice in the above two groups experienced no significant recurrence within 60 days. In contrast to the 20‐day median survival of those on group I, the median survival times of mice treated with anti‐PD‐L1 (group II), iMWA + Glue (group III) and iMWA + Glue + anti‐PD‐L1 (group IV) were 28, 26, and 30 days, respectively (Figure 7D). Overall, the novel combination therapy of injecting a cholesterol‐catalyzed hydrogel into the residual tumor after iMWA combined with an anti‐PD‐L1 antibody effectively inhibited the growth of both residual primary and distant tumors.

To further study the mechanism by which this cholesterol‐catalyzed hydrogel effectively enhances the immune efficacy of anti‐PD‐L1 after iMWA, we carefully evaluated the effect of this combination therapy on the immune system at 4 days after the last injection of anti‐PD‐L1. The results showed that intratumoral injection of the cholesterol‐catalyzed hydrogel after iMWA, with or without anti‐PD‐L1 injection, significantly promoted the maturation of DCs inside the lymph nodes adjacent to the primary tumors (Figure 7E; Figures S9,S12 and S13A, Supporting Information), consistent with its ability to induce HMGB1 release and CRT expression. Additionally, combination therapy with the cholesterol‐catalyzed hydrogel plus an anti‐PD‐L1 antibody after iMWA significantly increased the frequency of tumor‐infiltrating CD3^+^CD8^+^ cells inside distant tumors (Figure 7F; Figures S10,S12 and S13B, Supporting Information) and reduced the invasion of immunosuppressive T regulatory cells (Tregs) (Figure 7G; Figures S11,S12 and S13C, Supporting Information), significantly increasing the ratio of CD3^+^CD8^+^/Tregs in distant tumors (Figure 7H). Furthermore, the secretion levels of cytotoxic cytokines, including TNF‐α and IFN‐γ, in distant tumors increased significantly after combination therapy (Figure 7I,J), thus effectively inhibiting proliferation (Figures S14 and S15, Supporting Information) and enhancing the apoptosis of distant tumors (Figures S16 and S17, Supporting Information). Taken together, the combination therapy of cholesterol‐catalyzed hydrogel fixation and anti‐PD‐L1 injection in residual tumors after iMWA enhances the host antitumor immune response.

### 2.5 Safety Evaluation of Cholesterol‐Catalyzed Hydrogel‐Assisted iMWA Antitumor Therapy

To clarify the toxicity of cholesterol‐catalyzed hydrogels combined with iMWA for antitumor therapy, we carefully evaluated the combination therapy's biosafety using standard serum biochemical assays, routine complete blood analysis, and histological examination. Mice were sacrificed at 0, 12, 72, and 168 h after sequential treatment with iMWA or cholesterol‐catalyzed hydrogels and subsequently tested accordingly (Figure S18A, Supporting Information). First, microscopic observation of H&E‐stained sections of the main organs collected from mice at 0, 12, 72, and 168 h after combined treatment revealed no obvious histological damage (Figure S18B, Supporting Information). Second, after 12 h of combined treatment, the white blood cell (WBC) count increased significantly. The platelet (PLT), hemoglobin (HGB), mean corpuscular hemoglobin (MCH), mean corpuscular hemoglobin concentration (MCHC), and lymphocyte (LYM) count increased slightly, and the mean corpuscular volume (MCV) and monocyte (MON) and neutrophil (NEU) counts decreased slightly; however, these physiological indices gradually returned to their corresponding normal levels after 168 h of combined treatment. Moreover, the effects of combination therapy on the levels of red blood cells (RBCs), hematocrit (HCT), and eosinophils (EOS) were negligible (Figure S19, Supporting Information). Therefore, using cholesterol‐catalyzed hydrogels assisted by iMWA for antitumor therapy is unlikely to produce obvious side effects at the experimental dose.

## 3 Discussion

Cholesterol metabolism is crucial in shaping the immunosuppressive tumor microenvironment. Tumor cells require large amounts of cholesterol for rapid proliferation and membrane biogenesis, and its accumulation induces endoplasmic reticulum stress, leading to CD8^+^ T‐cell exhaustion and weakened antitumor activity.^[^ ^10^, ^22^ ^]^ Excess cholesterol also attracts suppressive immune cells (such as regulatory T cells and myeloid‐derived suppressor cells), promoting tumor immune evasion, and inhibits dendritic cell migration and maturation, reducing antigen presentation efficiency and suppressing host immune response.^[^ ^23^ ^]^ These mechanisms collectively contribute to the high recurrence and metastasis rates of cancers such as HCC.^[^ ^24^ ^]^ Removing cholesterol from the tumor microenvironment can significantly improve the immunosuppressive state, restore CD8^+^ T‐cell function, enhance the killing ability of tumor cells, reduce the aggregation of suppressive immune cells, promote the infiltration and activation of effector immune cells, and increase the efficiency of dendritic cell antigen presentation.^[^ ^25^ ^]^ Through these mechanisms, cholesterol clearance not only inhibits tumor recurrence and metastasis but also enhances the efficacy of immunotherapy, providing a novel strategy for treating tumors like HCC.

This study is the first to propose the use of cholesterol‐targeting catalytic hydrogels (DA‐COD‐OD‐HCS) to reverse immunosuppression in the tumor microenvironment and effectively improve the synergistic therapeutic effect of iMWA and ICIs. The novel injectable cholesterol‐catalyzed hydrogel system mainly includes DA‐COD, hemin, OD, and CS. First, DA‐COD can release COD in response to an acidic tumor microenvironment by stabilizing the enzyme activity of COD. Dimethyl maleic anhydride is an effective protein protective reagent used to decompose and recombine prokaryotic and eukaryotic ribosomes.^[^ ^18^, ^26^ ^]^ Dimethyl maleic acid introduces a negatively charged residue in place of the positive amino group, which causes electrostatic destabilization of the modified protein‐containing particles to release the protein under acidic conditions, as well as the reconstitution of the original particles from the dissociated components.^[^ ^18^, ^26^, ^27^ ^]^

The COD released by DA‐COD in the acidic tumor microenvironment can efficiently decompose cholesterol in iMWA residual tumor debris into 4‐cholestene‐3‐one and H_2_O_2_, thus alleviating the immunosuppressive effect of cholesterol on tumors. COD is a flavoprotein oxidoreductase with good catalytic activity for cholesterol and can oxidize cholesterol to 4‐cholestene‐3‐one and H_2_O_2._^[^ ^13^ ^]^ 4‐Cholestene‐3‐one has certain antitumor efficacy and can promote the release of HMGB1 from the nucleus to the cytoplasm of tumor cells, block the nuclear translocation of HIF‐1α, and inhibit the activation of MMP‐2 and MMP‐9, thus inhibiting the invasion and metastasis of tumors.^[^ ^14^ ^]^ Additionally, H_2_O_2_, another cholesterol decomposition product catalyzed by COD, can react with hemin in the Fenton reaction. Hemin, reported to be an effective Fenton catalyst, can induce continuous lipid peroxidation in the presence of a Fe^2+^‐based catalyst through the production of ·OH mediated by the Fenton reaction, thus leading to effective ferroptosis in tumors, which can further improve antitumor immune efficacy.^[^ ^16^, ^28^ ^]^ Interestingly, hemin can be covalently coupled with CS through an amide formation reaction. Thus, we mixed hemin and CS to prepare highly water‐soluble HCSs.

OD and CS were used in this study to demonstrate the long‐term remodeling effect of COD on the immune microenvironment of iMWA‐treated residual tumors. OD and CS rapidly formed gels and fixed COD and hemin in tumors, providing favorable conditions for the long‐term anti‐liver cancer efficacy of iMWA sequential ICI treatment. Dex and CS are two kinds of polysaccharides widely used in hydrogel preparation because of their good biocompatibility and biodegradability.^[^ ^29^ ^]^ Dex is rich in ortho‐hydroxyl groups, which can be oxidized to aldehydes (‐CHO) and form Schiff base bonds with ‐NH_2._^[^ ^29^, ^30^ ^]^ In this study, Dex was oxidized to OD by sodium periodate. In addition, the ‐NH_2_ groups of CS can react with ‐COOH and ‐CHO to form amide and Schiff base bonds, respectively.^[^ ^29^, ^30^, ^31^ ^]^ Therefore, under the activation of EDC and NHS, a double network gel containing amide bonds and dynamic Schiff base bonds was constructed from OD and CS in this study; this gel has good elasticity, high expansibility, and strong tissue adhesion in the residual tumor microenvironment.

The cholesterol‐targeting catalytic hydrogel DA‐COD‐OD‐HCS designed in this study uses tumor fragments generated by iMWA as fuel to effectively inhibit the growth of residual tumors by inducing sustained lipid peroxidation, which has been demonstrated in the treatment of 4T1 and H22 mouse tumors as well as in situ N1S1 liver cancer models in SD rats. Moreover, DA‐COD‐OD‐HCS hydrogel‐mediated DAMPS exposure after iMWA treatment initiated host‐specific antitumor immunity by promoting effective DC maturation, CD8^+^ T‐cell infiltration, and cytotoxic cytokine secretion. Studies have shown that free cholesterol in the tumor microenvironment can induce CD8+ T‐cell apoptosis under ER stress and inhibit DC maturation and migration to draining lymph nodes, ultimately significantly reducing the tumor response to ICIs.^[^ ^32^ ^]^ Thus, combined with anti‐PD‐L1 immunotherapy, iMWA treatment combined with sequential intratumoral injection of the DA‐COD‐OD‐HCS hydrogel immobilizes COD for a long period, facilitating the removal of cholesterol from the residual tumor, effectively inhibiting the growth of the primary tumor and distant metastases without potential side effects in adjacent and distant tissues.

In summary, this study focused on the residual tumor microenvironment after iMWA and proposed an innovative antitumor strategy in which a cholesterol‐catalyzed hydrogel using tumor debris as fuel led to the targeted removal of cholesterol, the main component of the immunosuppressive residual tumor microenvironment. In addition, cholesterol‐catalyzed hydrogels have many advantages, such as good biocompatibility, ease of use, and enhanced synergistic therapeutic effects when combined with MWA and ICIs, indicating substantial potential for clinical application in the treatment of HCC and other tumors.

## 4 Chemicals and Materials

DA, SA, sodium periodate, and 1‐ethyl‐(3‐dimethylaminopropyl) carbodiimide hydrochloride (EDC) were purchased from Shanghai Aladdin Biochemical Technology Co., Ltd. COD and CS were purchased from Shanghai Yuanye Biotechnology Co., Ltd. Dextran‐4 was purchased from Serva. N‐hydroxysuccinimide (NHS) was purchased from Alorich. Hemin and DCFH‐DA were purchased from Sigma Aldrich. BODIPY 581/591 C11 was purchased from Thermo Fisher. Anti‐PD‐L1 (catalog: BE0101) was purchased from BioXcell. Sodium bicarbonate (NaHCO_3_) was obtained from China National Pharmaceutical group Chemical Reagent Co., Ltd. RPMI 1640 cell culture medium was purchased from Cyclone Laboratories, Inc. DMEM high glucose cell culture medium (Gibco) was purchased from Thermo Fisher Scientific, and fetal bovine serum was purchased from Bovogen Biological Products Co., Ltd. Anti‐HMGB1 antibody (catalog: 70‐ab40050‐100) was obtained from MultiSciences. Anti‐CRT antibodies (catalog: ab2907) were obtained from Abcam. The Alexa‐488‐conjugated secondary antibody (catalog: 111‐545‐003) was obtained from Jackson. Antibodies used for flow cytometry, including anti‐CD3‐FITC (Biogene, clone 17A2, catalog: 100 204), anti‐CD4‐APC (Biogene, clone GK1.5, catalog: 100 412), anti‐CD8‐PE (Biogene, clone 53–6.7), catalog: 100 708), and anti‐Foxp3‐PE (Biogene, clone MF‐14, catalog: 126 404), anti‐CD11c FITC (Biogene, clone N418, catalog: 117 306), anti‐CD80‐PE (Biogene, clone 16‐10A1, catalog: 104 708) and anti‐CD86‐APC (Biogene, clone GL‐1, catalog: 105 012), were obtained from Biogene or eBioscience and diluted 1:300 for cell staining.

## 5 Experimental Section

### Preparation and Characterization of Cholesterol‐Targeted Catalytic Hydrogels

*Modification of COD*: COD (5 mg mL^−1^), DA, and SA (both 2.5 mg mL^−1^) were fully dissolved in pH 9.0 HEPES buffer and stirred at room temperature for 3–4 h. DA‐COD and SA‐COD were purified by a centrifugal filter (MWCO = 10 kDa) and preserved at −20 °C.

*Preparation of HCS*: The carboxyl group of Hemin was activated in anhydrous DMSO with a molar ratio of 1:1:1 (Hemin: EDC: NHS) for 2 h. CS was then mixed with activated Hemin at a molar ratio of 5:1 and stirred overnight. HCS was obtained by soaking the mixture in sodium bicarbonate solution (pH 8–9) for 24 h, followed by freeze‐drying for 72 h in a dialysis bag (MWCO = 8000–14 000) and storage at −20 °C.

*Preparation of OD*: Dextran‐4 (111.7 mg mL^−1^) and sodium periodate (110.6 mg mL^−1^) were mixed in ddH_2_O, stirred at room temperature in the dark for 72 h, purified in a dialysis tube (MWCO = 3500) for 48 h, and freeze‐dried to obtain OD, which was subsequently stored at −20 °C.

*Preparation and assessment of the DA‐COD‐OD‐HCS hydrogel*: OD at 10, 20, and 30 wt.% and HCS at 1, 5, and 10 wt.% were mixed, photographed, and observed at 0.5, 5, and 20 min. The rheological properties of the OD, HCS, and OD‐HCS hydrogels were confirmed by rotary rheometry (Haake Rheo Stress 6000, Germany, PP20H, frequency set to 10 rads^−1^), and their elastic modulus (G’) and viscous modulus (G’’) were measured. The morphologies of the OD, HCS, and DA‐COD‐HCS‐OD hydrogels were observed by scanning electron microscopy (SEM, Zeiss Supra 55). Finally, 20 wt.% OD, 5 wt.% HCS and 50 U mL^−1^ COD, DA‐COD, or SA‐COD were stirred for 5 min at room temperature to prepare the COD‐OD‐HCS, DA‐COD‐OD‐HCS, or SA‐COD‐OD‐HCS hydrogels. These hydrogels were labeled with 2 mg mL^−1^ Cy5.5 dye as described above.

### Catalytic Activity of the Cholesterol‐Targeted Catalytic Hydrogel

An ABTS‐HRP enzyme activity assay was used to quantify the generated H_2_O_2_ to evaluate the effectiveness of DA‐COD‐OD‐HCS hydrogel‐mediated hydrogen peroxide generation.^[^ ^19^ ^]^ First, 50 µL of hydrogen peroxide solution at different concentrations (0, 100, 200, 300, and 400 µM) was added to each well of a 96‐well plate, after which 150 µL of ABTS‐HRP detection solution was added and allowed to react for 5 min. The OD values were measured at 800 nm by a UV‒vis spectrophotometer, and an H_2_O_2_ standard curve was drawn. Subsequently, 5 µL of cholesterol (CHOL, 50 µM), linoleic acid (LA, 450 µg mL^−1^), or cancer cell lysate (1 × 10^6^ cells mL^−1^) was added to 495 µL of 0.5 U mL^−1^ COD, DA‐COD or SA‐COD with or without OD‐HCSs for 5 min at pH 6.8 or 7.4, respectively. Then, 50 mL of each reaction mixture was added to each well of the 96‐well plate, and 150 µL of ABTS‐HRP detection solution was added for 5 min. Finally, the OD value of the mixture at 800 nm was determined by a UV‒vis spectrophotometer, and the cholesterol‐catalyzing activity was determined according to the above H_2_O_2_ standard curve.

Each sample (1 U mL^−1^) was incubated at pH 6.8 or pH 7.4 for 2, 4, 8, 12, or 24 h to evaluate the effect of different pH values on the enzyme catalytic activity of COD, DA‐COD, or SA‐COD. The cholesterol‐catalyzing ability of each sample at different pH values was determined using the ABTS‐HRP assay.

A mixture of TMB (1 mM), COD/DA‐COD/SA‐COD (5 U mL^−1^), or OD‐HCS ([hemin] = 20 µg mL^−1^) was extruded by an injection syringe into a mixture containing cholesterol (5 mg mL^−1^) or cell lysate (1 × 10^6^ dead cells) to evaluate the ability of the catalytic hydrogel to produce •OH. Optical images were recorded 5 min after the injection.

### Cell Experiment

Mouse 4T1 breast cancer cells (SCSP‐5056), mouse CT26 colon cancer cells (TCM37), human HeLa cervical cancer cells, human A549 lung cancer cells, and mouse H22 liver cancer cells (ZQ0109) were obtained from the Key Laboratory of Imaging Diagnosis and Minimally Invasive Interventional Research of Zhejiang Province. No mycoplasma contamination was found in any of the cell lines before use.

H22 cells (1 × 10^5^ cells per well) preinoculated in 12‐well plates were incubated with PBS, COD, DA‐COD, or SA‐COD in the presence or absence of cell lysate (1 × 10^6^ dead cells per well) for 6 h to evaluate the ability of cholesterol‐catalyzed hydrogels to induce intracellular lipid peroxidation. The pH was adjusted to 7.4 or 6.8. Subsequently, the cells in each group were washed with PBS and incubated in fresh culture solution containing BODIPY‐C11 (1.5 µM) or DCFH‐DA dye (1.5 µM) for 30 min. Then, the cells were fixed with 4 wt.% paraformaldehyde solution, stained with 1 µg mL^−1^ DAPI, and imaged with a laser confocal microscope (Leica, STELLARIS5). In addition, H22 cells in each group were incubated in fresh medium containing BODIPY‐C11 (20 µM) or DCFH‐DA (20 µM) for 4 h, detected by flow cytometry (BD, FACSCanto II), and analyzed using FlowJo_v10.8.1 (FlowJo Software).

H22 cells preinoculated in 12‐well plates (1 × 10^5^ cells per well) and cell lysate (1 × 10^6^ dead cells per well) were incubated for 6 h in the presence or absence of Fer‐1 (10 µM) and GSH (1 mM) to evaluate the rescuing effect of Fer‐1 and GSH. The cells were then stained with BODIPY‐C11 (1.5 µM) dye and observed by confocal microscopy.

H22 cells were treated as described above for 24 h to evaluate the HMGB1 release profile. The cells were then washed twice with PBS, fixed in 4 wt.% paraformaldehyde solution for 20 min, permeabilized with 0.1 wt.% Triton X‐100 for 10 min, blocked with 5% FBS for 30 min, and stained with primary anti‐HMGB1 antibody (1:1000) for 1 h and Alexa‐525 conjugated secondary antibody (1:500) for 30 min following the manufacturer's procedure. The cells were then counterstained with DAPI for 10 min and observed using confocal microscopy. The HMGB1 release profiles of these H22 cells after various treatments were evaluated by flow cytometry.

H22 cells presided in 12‐well plates (1 × 10^5^ cells per well) received the same treatments as described above and were subsequently sequentially stained with the primary anti‐CRT antibody (1:1000) for 1 h, Alexa‐488‐conjugated secondary antibody (1:500) for 30 min, and DAPI for 10 min to evaluate the calreticulin (CRT) expression profile. The counterstained cells were subjected to confocal microscopic observation and flow cytometric analysis.

To evaluate the capacity of the catalytic hydrogel to induce cell death, H22, 4T1, HeLa and A549 cells preseeded in 12‐well plates (1 × 10^5^ cells per well) were incubated with COD‐OD, SA‐COD‐OD, or DA‐COD‐OD in the presence or absence of the corresponding cell lysates (1 × 10^6^ dead cells per well) for 24 h before their cell viabilities were determined using the standard MTT assay.^[^ ^19^, ^20^ ^]^

### Animal Experiment

Female BALB/c mice and male SD rats were purchased from Hangzhou Hangsi Biotechnology Co., Ltd., and used according to the protocol approved by the Experimental Animal Center of Lishui Central Hospital. H22 and 4T1 cells (2 × 10^6^) suspended in 50 µL of PBS were subcutaneously injected into the right flank of each BALB/c mouse to establish subcutaneous H22 and 4T1 tumor models. N1S1 cells (6 × 10^6^) were mixed into 100 ml PBS containing 30% matrix adhesive (Corning) and injected into the right lower hepatic lobe of each SD rat to establish a liver in situ N1S1 tumor model. Efforts were made to reduce the animals’ pain

### Intratumoral Retention of the DA‐COD‐OD‐HCS Hydrogel Post iMWA

iMWA was performed by inserting a microwave ablation probe sterilized with 75% ethanol 0.5 cm into mouse tumors, adjusting the output power of microwave ablation to 5 W and time to 2 min. The surface temperature of the tumor was monitored using an infrared thermal imager (Fortric225), and the temperature of the tumor was maintained below 60 °C.^[^ ^20^, ^21^ ^]^

To evaluate the retention effect of the DA‐COD‐OD‐HCS hydrogel after iMWA of the tumor, we carefully recorded the retention time of the Cy5.5‐labeled DA‐COD‐OD‐HCS hydrogel (10 mg mL^−1^) after iMWA (5 W, 2 min) in the tumor. Six H22 tumor‐bearing mice were randomly divided into two groups and irradiated by microwave for 5 W and 2 min. Then, three mice were injected with Cy5.5‐labeled DA‐COD‐OD‐HCS hydrogel (10 mg mL^−1^; gel group), and three were injected with Cy5.5‐labeled DA‐COD (10 mg mL^−1^; without glue group). The mice were subsequently assessed with an in vivo imaging system (IVIS, PerkinElmer) at different time points (0.5, 24, 48, 72, 120 h) after injection, and the fluorescence intensity of Cy5.5 in each group was recorded. Another twenty‐four H22 tumor‐bearing mice in each group (n = 6) were injected with DA‐COD + OD‐HCS (group I), DA‐COD + OD (group II), DA‐COD + HCS (group III), or DA‐COD (group IV) sequentially after iMWA. Finally, the tumor tissues were collected at 24 h and 72 h after injection, and the frozen sections were observed under a confocal microscope.

To evaluate the ability of cholesterol‐catalyzed hydrogels to induce intracellular lipid peroxidation in vivo, we evaluated HMGB1 release profiles and lipid peroxidation in the tissues of H22 tumor‐bearing mice after different treatments. Twelve H22 tumor‐bearing mice were randomly divided into 4 groups (n = 3): group I, iMWA; group II, iMWA + Glue; group III, iMWA + DA‐COD + Glue; and group IV, iMWA + SA‐COD + Glue. Glue indicates the OD‐HCS hydrogel. All groups were treated with iMWA, and the residual tumors in groups II, III, and IV were injected with the OD‐HCS, DA‐COD‐OD‐HCS, and SA‐COD‐OD‐HCS hydrogels, respectively. After 24 h, the tumor sections from each group were incubated with an anti‐HMGB1 primary antibody (1:200) for 1 h and then incubated with an Alexa‐525‐conjugated secondary antibody (1:500). Finally, the sections were counterstained with DAPI for 10 min and observed using laser confocal microscopy. In addition, the tumor tissue sections were incubated with BODIPY‐C11 dye (1.5 µM) for 30 min, counterstained with DAPI for 10 min, and observed using confocal microscopy.

### In Vivo Cancer Treatment

H22 or 4T1 tumor‐bearing mice (≈80 mm^3^ in volume) were randomly divided into six groups (n = 6 per group) to investigate the antitumor effect of different treatment methods on tumor‐bearing mice (group I, Ctrl; group II, iMWA; group III, iMWA + Glue+ SA‐COD; group IV, DA‐COD; group V, DA‐COD + Glue; group VI, iMWA + Glue + DA‐COD. Glue indicates OD‐HCSs). In group I, H22 or 4T1 tumor‐bearing mice were directly injected with 100 µM BBT‐2FT probe. The H22 or 4T1 tumor‐bearing mice in groups II and VI were first treated with iMWA (5 W, 2 min), and then the residual tumors in groups III and VI were injected with BBT‐2FT‐labeled SA‐COD‐OD‐HCS (10 mg mL^−1^) and BBT‐2FT‐labeled DA‐COD‐OD‐HCS (10 mg mL^−1^), respectively. Groups IV and V were directly injected with BBT‐2FT‐labeled DA‐COD (10 mg mL^−1^) and BBT‐2FT‐labeled DA‐COD‐OD‐HCS (10 mg mL^−1^), respectively. Then, the mice in each group were observed using an NIR‐II in vivo imaging system (MARS, Artemis Intelligent Imaging) at different time points (0, 7, 14, and 21 days) after injection, and the NIR‐II fluorescence intensity of the BBT‐2FTs in each group was recorded at the same time. At the beginning of treatment, the length and width of the tumors in each group were recorded with a digital caliper every other day, and the tumor volume was calculated according to the following formula: tumor volume = L*W*W/2. Death was defined as a tumor volume greater than 1000 mm^3^, and the survival time of each mouse was recorded. A digital balance was used to record the weight of each mouse throughout the treatment. After the mice were killed, the tumors from each group were embedded in paraffin, cut into sections, and stained with hematoxylin and eosin (H&E) and TUNEL to evaluate the antitumor effect in each group.

Twenty‐four N1S1 tumor‐bearing SD rats were randomly divided into four groups (n = 6) to further verify the efficacy of MWA combined with a cholesterol‐catalyzed hydrogel in the treatment of an orthotopic N1S1 tumor model in rats: group I, Ctrl; group II, iMWA + SA‐COD + Glue; group III, iMWA + COD + Glue; and group IV, iMWA + DA‐COD + Glue. On day 0, groups II, III, and IV were first treated with iMWA. The MWA probe was disinfected with 75% ethanol and inserted into the N1S1 liver tumor in situ, and the MWA output power was set to 5 W for 2 min. An infrared thermal imager (Fortric225) was used simultaneously to monitor the surface temperature of the tumor, and the tumor temperature was maintained below 60 °C. Within ten minutes after iMWA, groups II, III, and IV were injected with SA‐COD + Glue, COD + Glue, and DA‐COD + Glue, respectively. Glue indicates OD‐HCSs. Subsequently, 3.0‐T MRI imaging was performed on the SD rats in each group on days 0, 7, and 14, and the tumor volume in each group was recorded and quantified. Finally, after the rats in each group were sacrificed, the orthotopic liver tumors were extracted for H&E and Ki67 immunohistochemical staining.

### Assessment of the Mechanism by Which Cholesterol‐Catalyzed Hydrogels Enhance Antitumor Immunotherapy In Vivo

A double tumor mouse model of orthotopic and distant tumors was established to explore the mechanism by which MWA combined with the DA‐COD‐OD‐HCS hydrogel enhances antitumor immunotherapy. 4T1 cells (2 × 10^6^) suspended in 50 µL of PBS were subcutaneously injected into the right and left flanks of mice on days 0 and 7, representing primary and distant tumors, respectively. On day 8, 30 mice were randomly divided into six groups (n = 5) and treated as follows: group I, Ctrl; group II, anti‐PD‐L1; group III, iMWA + Glue; group IV, iMWA + Glue + anti‐PD‐L1; group V, iMWA + Glue + DA‐COD; and group VI, iMWA + Glue + DA‐COD + anti‐PD‐L1. Glue indicates the OD‐HCS hydrogel. Each mouse in group II was injected with an anti‐PD‐L1 antibody (20 µg time^−1^) through the tail vein. The primary tumors on the right flank of the mice in groups III, IV, V, and VI were first treated with iMWA. Subsequently, the OD‐HCS hydrogel (10 mg mL^−1^) was injected into the right residual tumor of group III mice. In group IV, the OD‐HCS hydrogel (10 mg mL^−1^) was injected into the right residual tumor, followed by anti‐PD‐L1 (20 µg time^−1^) into the tail vein. In group V, the right residual tumor was injected with the DA‐COD‐OD‐HCS hydrogel (10 mg mL^−1^). In group VI, the DA‐COD‐OD‐HCS hydrogel (10 mg mL^−1^) was injected into the right residual tumor, followed by anti‐PD‐L1 (20 µg time^−1^) into the tail vein. Additionally, all mice in groups II, IV, and VI were injected with anti‐PD‐L1 (20 µg time^−1^) via the tail vein on days 9, 11, and 15. The length and width of each tumor were recorded with digital calipers every other day throughout the treatment, and the tumor volume was calculated according to the following formula: tumor volume = L*W*W/2. A tumor volume larger than 1000 mm^3^ was defined as death, and the survival time of each group was recorded in detail. A digital balance was used to record the weight of each mouse throughout the treatment.

Each group of mice was killed on the 19th day, the lymph nodes adjacent to the primary tumor and distant tumors were collected, and single‐cell suspensions and homogenates were prepared according to a well‐established protocol for subsequent antibody labeling. Then, according to the manufacturer's instructions, DCs (CD80^+^ CD86^+^) in the lymph nodes and CD8^+^ T cells (CD3^+^ CD4^−^ CD8^+^) and Tregs (CD3^+^ CD4^+^ Foxp3^+^) in the distant tumors were stained with the corresponding commercial fluorophore‐labeled antibodies and analyzed via flow cytometry. Additionally, after obtaining distant tumors from each group and generating paraffin sections, immunofluorescence antibodies against CD86 (BD Biosciences, catalog: 553 689), CD8 (Invitrogen Antibodies, catalog: 14‐0081‐82), Foxp3 (Invitrogen Antibodies, catalog: 14‐5773‐82), Ki67 (Abcam, catalog: ab15580) and TUNEL (Abcam, catalog: ab66108) were used to measure protein expression and localization after different treatments. The supernatant of the tumor homogenate was collected, and the concentrations of tumor necrosis factor‐α (TNF‐α) and interferon‐γ (IFN‐γ) were measured using TNF‐α (Invitrogen, catalog: 88‐7324‐88) and IFN‐γ (Invitrogen, catalog: 88‐7314‐88) ELISA kits.

### Safety Evaluation

Twelve healthy female mice were randomly divided into 4 groups (n = 3) to investigate the safety of MWA combined with the cholesterol‐catalyzed hydrogel treatment. Whole blood was collected from the orbit at 0, 12, 72, and 168 h after catalytic hydrogel injection, and routine blood indices (WBC, RBC, PLT, HGB, MCV, MCH, MCHC, HCT, MON%, EOS%, NEU%, LYM%) were tested. The main organs (heart, liver, spleen, lung, and kidney) were collected at the different points described above and sliced for H&E staining.

## Conflict of Interest

The authors declare no conflict of interest.

## Author Contributions

L.S., Z.Y., and Y.Z. contributed equally to this work. J.S.J., Z.L., C.Y.L., L.Z.F., L.C., and M.J.C. designed the study. J.S.J., L.S., Z.W.Z., Y.P.S., J.C.Y., Y.Z., Y.R.B., and G.F.S. performed data acquisition. L.S., X.X.C., Q.W.L., and J.Y.D. analyzed the data. Y.P.S., J.C.Y., and Y.Z. verified the data. L.S., Z.W.Z., and M.J.C. wrote the manuscript. G.F.S., Z.J.Y., and J.S.J. revised the manuscript. All authors read and approved the final manuscript.

## Supporting information



Supporting Information

## Data Availability

The data that support the findings of this study are available from the corresponding author upon reasonable request.
