# Supplementary material for: Cholesterol Targeted Catalytic Hydrogel Fueled by Tumor Debris can Enhance Microwave Ablation Therapy and Anti‐Tumor Immune Response
Source: Adv Sci (Weinh). 2024 Dec 12;12(5):2406975. doi: 10.1002/advs.202406975 (PMC11791989; doi:10.1002/advs.202406975)
Supplement: Supplementary file 1 — Supporting Information [file ADVS-12-2406975-s001.docx]

**Cholesterol-targeted catalytic hydrogels fueled by tumor debris can enhance microwave ablation therapy and the antitumor immune response**

**Running title:** Immune-sensitizing effect of cholesterol-targeted catalytic hydrogels on tumor microwave ablation

**Supplementary Figures:**

**
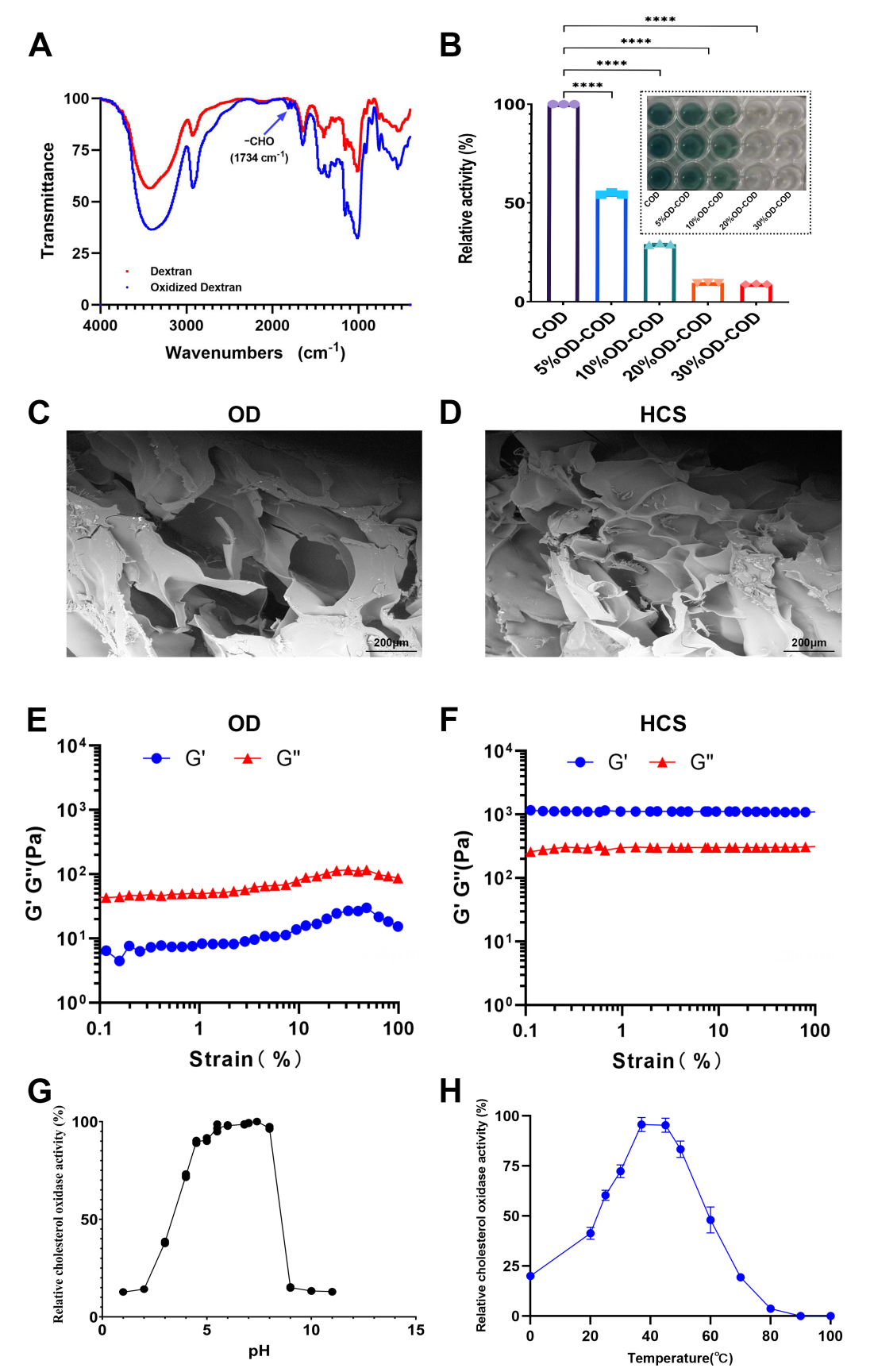
**

**Figure S1. (A)** FT-IR spectra of commercial dextran and OD. **(B)** HRP-ABTS assay demonstrating the effects of PBS and 5, 10, 20, and 30 wt.% OD on the relative cholesterol-catalytic activity of COD. Inset picture: HRP-ABTS detection and observation images of different groups. **(C, D)** SEM image of OD (C) and HCS (D). **(E, F)** G’ (storage modulus) and G’’ (loss modulus) of OD (E) and HCS (F) in strain sweep measurements. **(G)** Relative cholesterol-catalytic activity of COD under different pH conditions. **(H)** Relative cholesterol-catalytic activity of COD at different temperatures. The data in this figure are presented as the means ± SEMs, n = 3 biologically independent samples. *p < 0.05, **p < 0.01, ***p < 0.001, ****p < 0.0001.


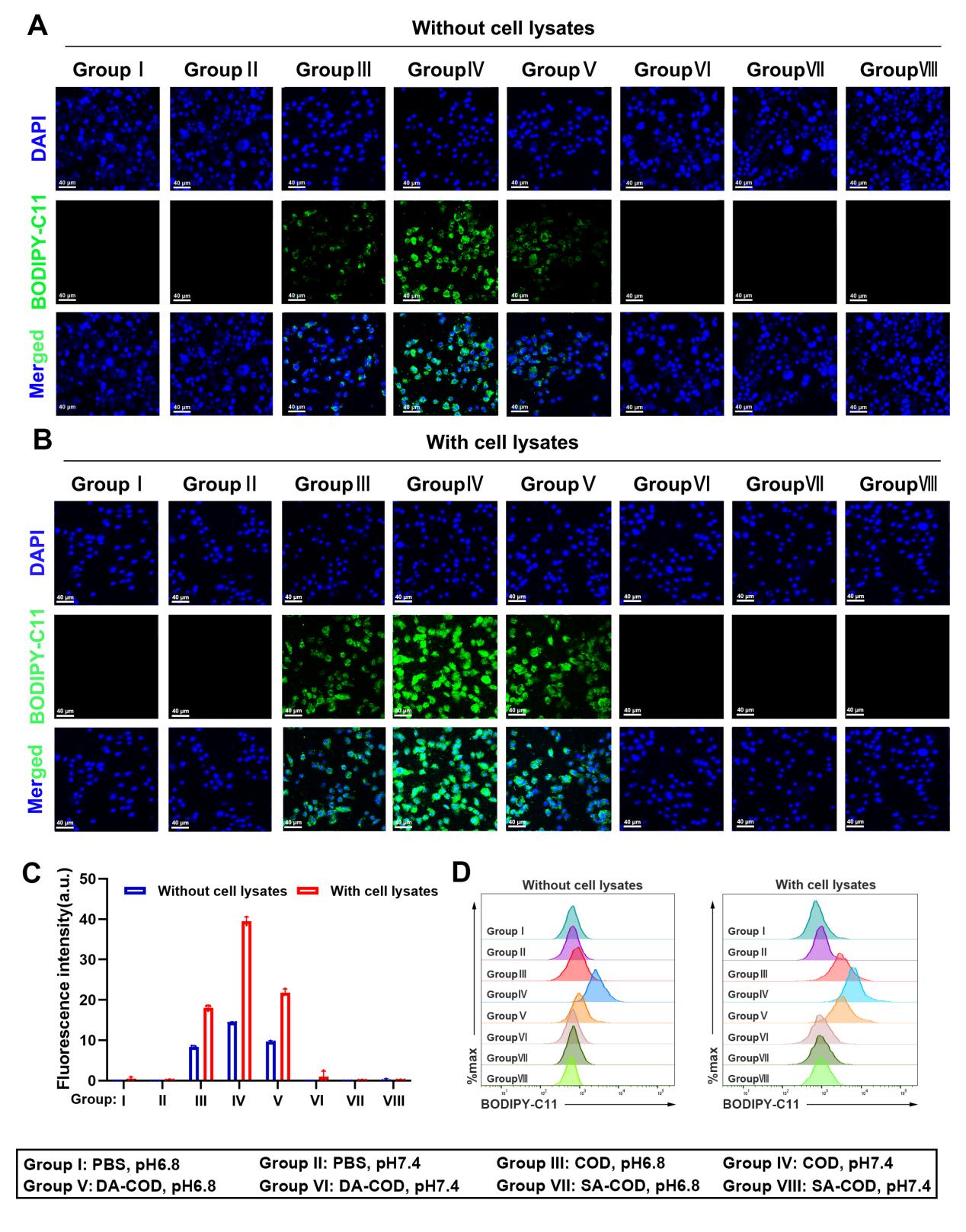


**Figure S2. (A, B)** Confocal imaging of the intracellular lipid peroxidation of H22 cells subjected to different treatments, as indicated, in the absence (A) or presence (B) of cell lysates stained with the BODIPY-C11 probe. **(C, D)** Semiquantitative analysis (C) and flow cytometry (D) of intracellular BODIPY-C11 fluorescence signals based on (A) and (B). The data are presented as the means ± SEMs; n = 3 biologically independent samples. *p < 0.05, **p < 0.01, ***p < 0.001, ****p < 0.0001.


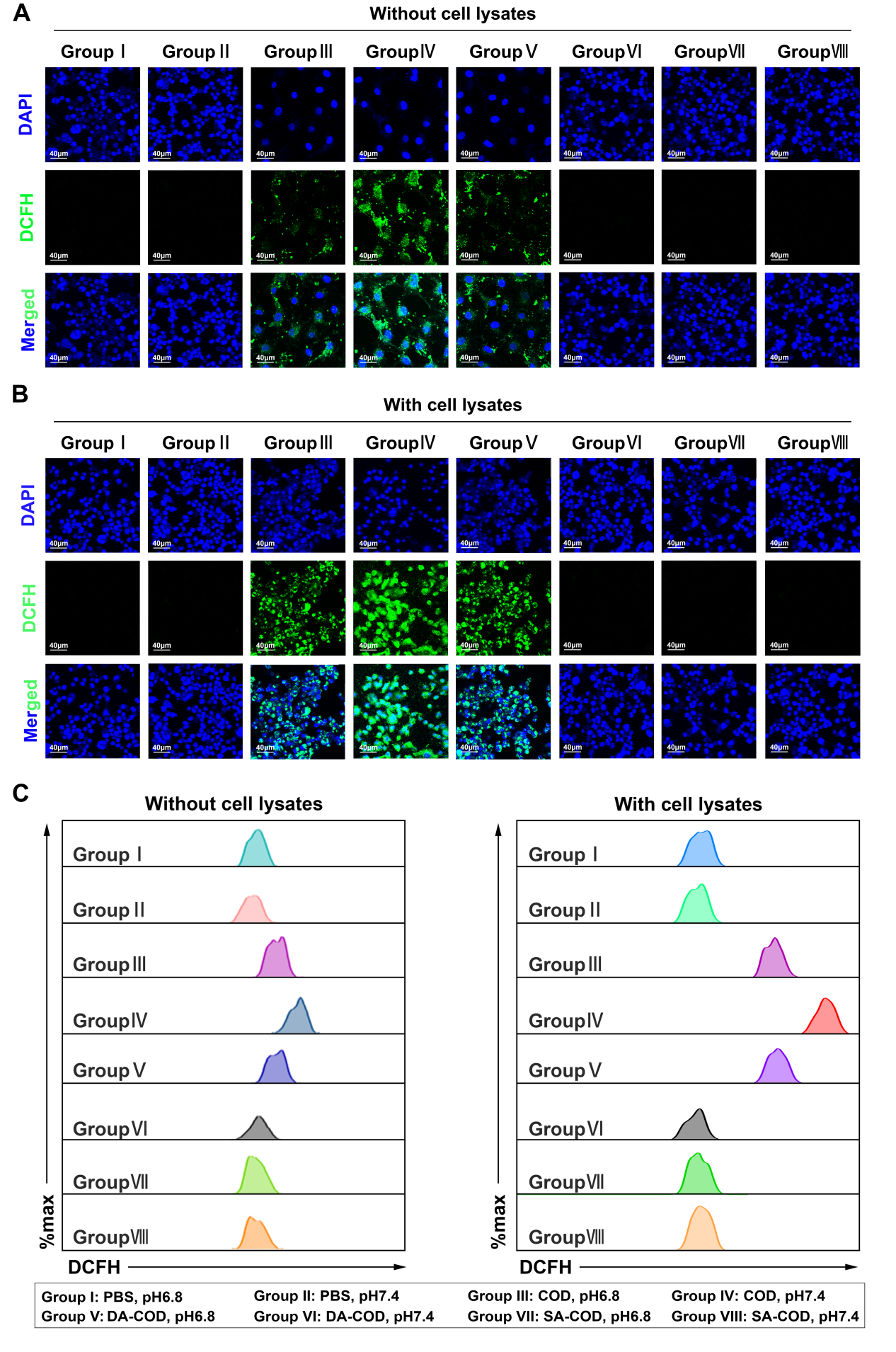


**Figure S3. (A, B)** Confocal imaging of the intracellular lipid peroxidation of H22 cells subjected to different treatments, as indicated, in the absence (A) or presence (B) of cell lysates stained with the probe DCFH-DA. **(C)** Flow cytometry of intracellular DCFH-DA fluorescence signals based on (A) and (B).


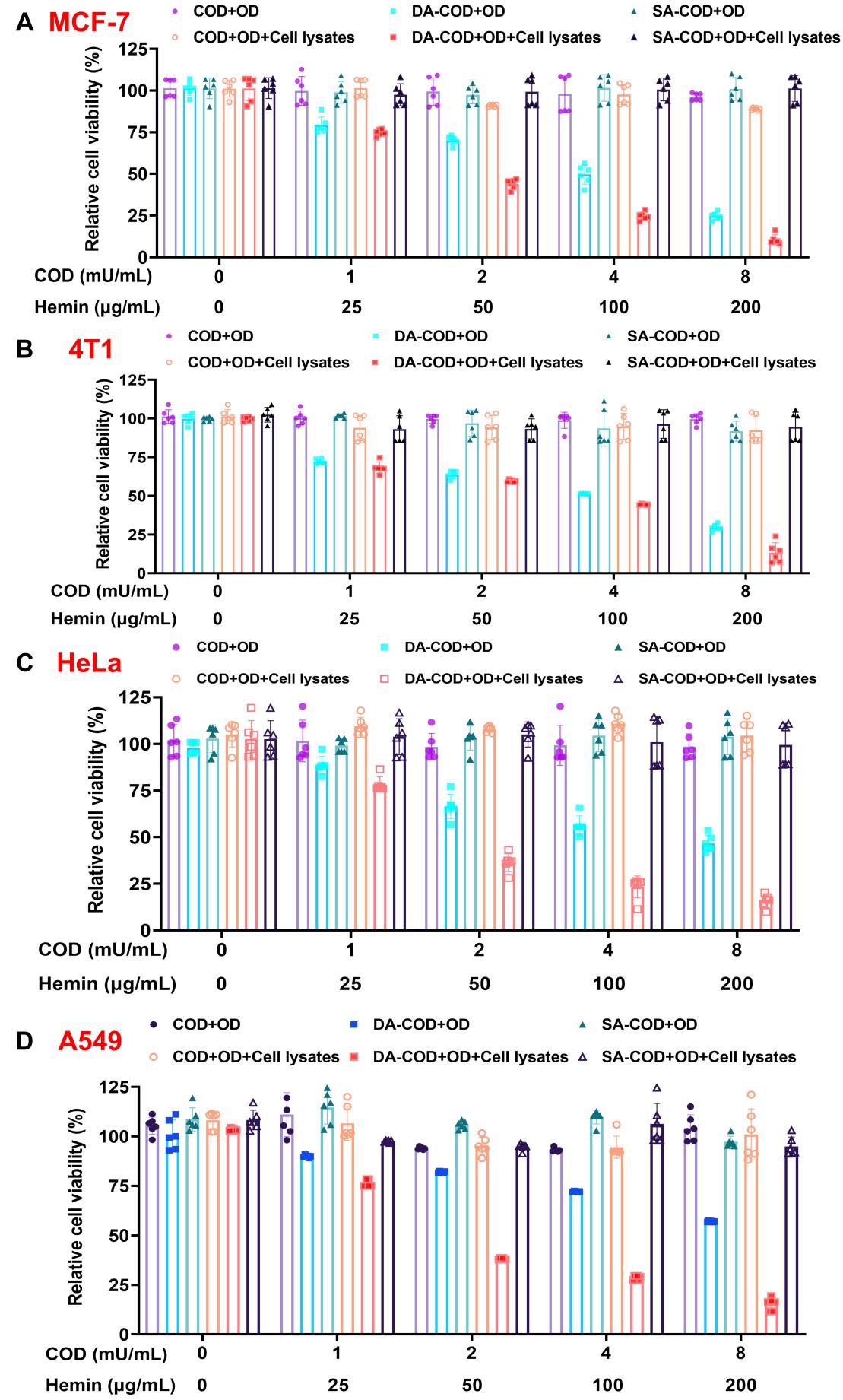


**Figure S4. (A-D)** Relative viabilities of MCF-7 (A), 4T1 (B), HeLa (C), and A549 (D) cells treated with the catalytic hydrogel in the presence or absence of lysate. The data are presented as the means ± SEMs; n = 6 biologically independent samples.

**
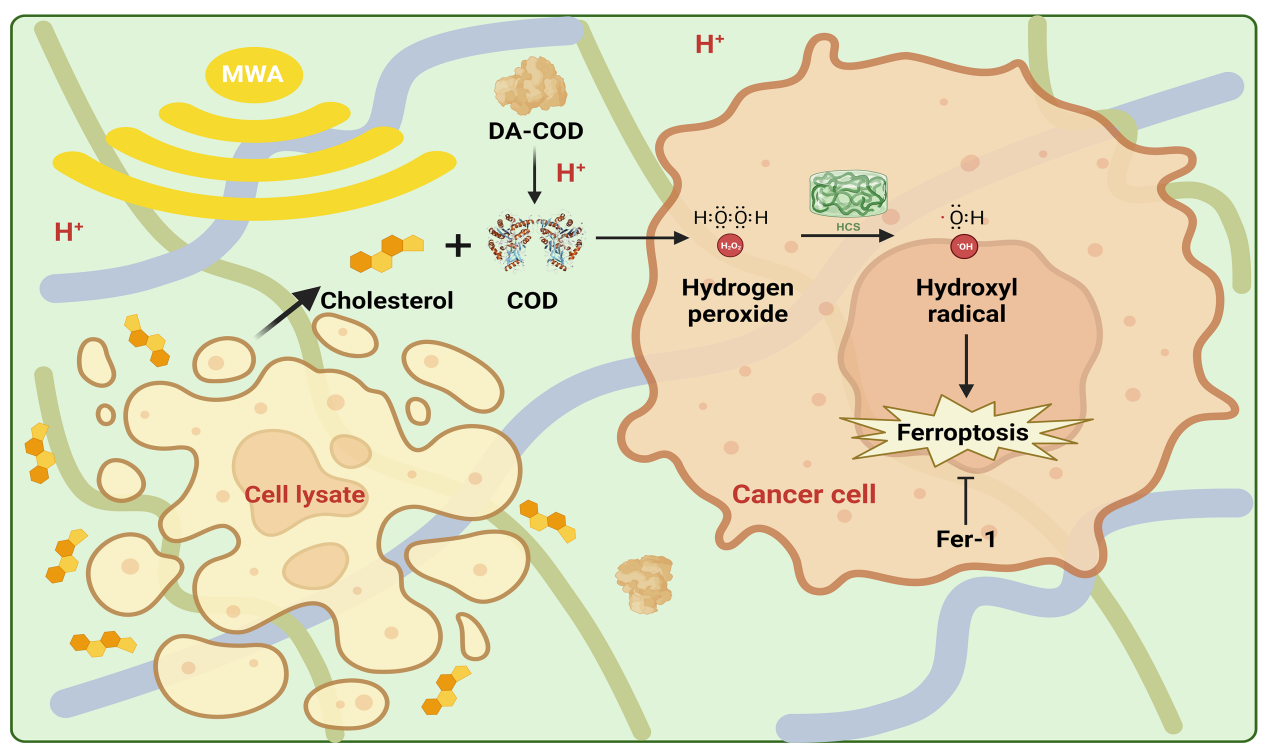
**

**Figure S5.** A schematic diagram illustrating the ferroptosis of cancer cells caused by DA-COD-OD-HCS hydrogels, which catalyze cholesterol in tumor debris after MWA.

**
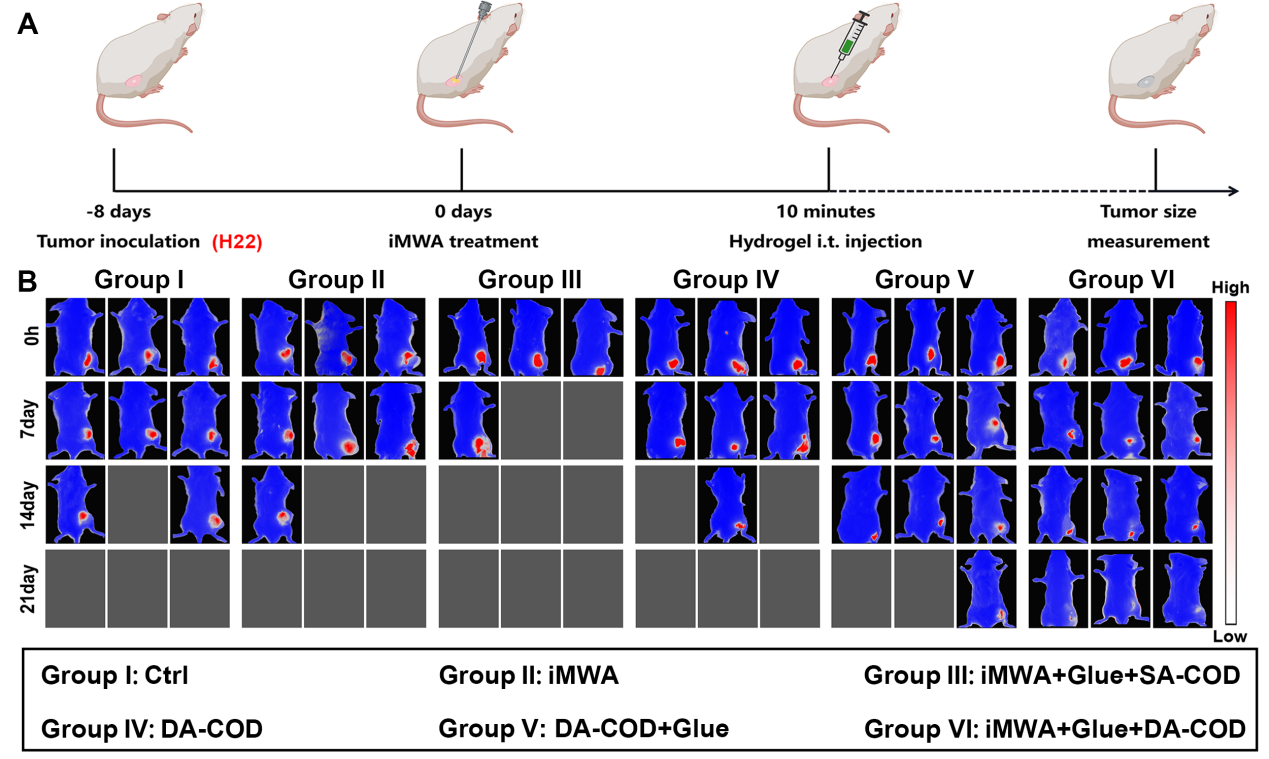
**

**Figure S6. (A)** Schematic illustration of the *in vivo* therapeutic schedule for the mouse H22 tumor model. **(B)** Representative *in vivo* NIR-II bioluminescence images of H22 tumor model mice after different treatments.

**
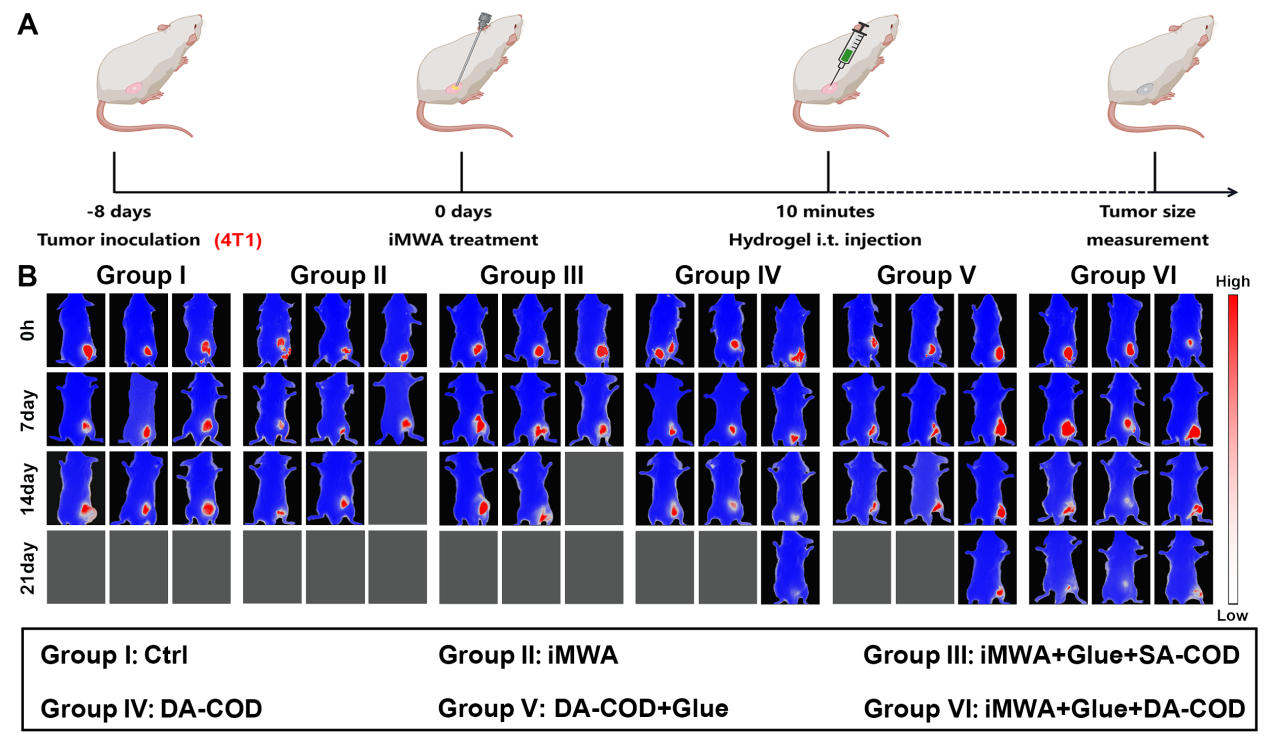
**

**Figure S7. (A)** Schematic illustration of the *in vivo* therapeutic schedule for the mouse 4T1 tumor model. **(B)** Representative *in vivo* NIR-II bioluminescence images of 4T1 tumor model mice after different treatments.


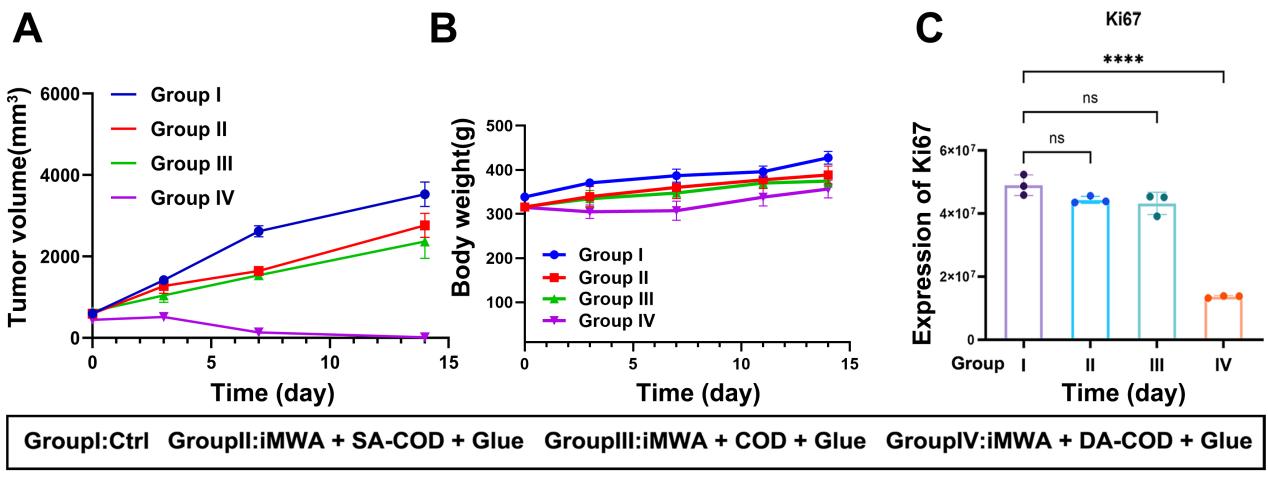


**Figure S8.** **(A)** Average tumor growth curve in different groups of N1S1-bearing rats after various treatments, as indicated. **(B)** Body weights of N1S1 tumor-bearing rats after various treatments. **(C)** Relative expression of Ki67 in tumor slices subjected to different treatments. Glue, OD-HCS hydrogel.


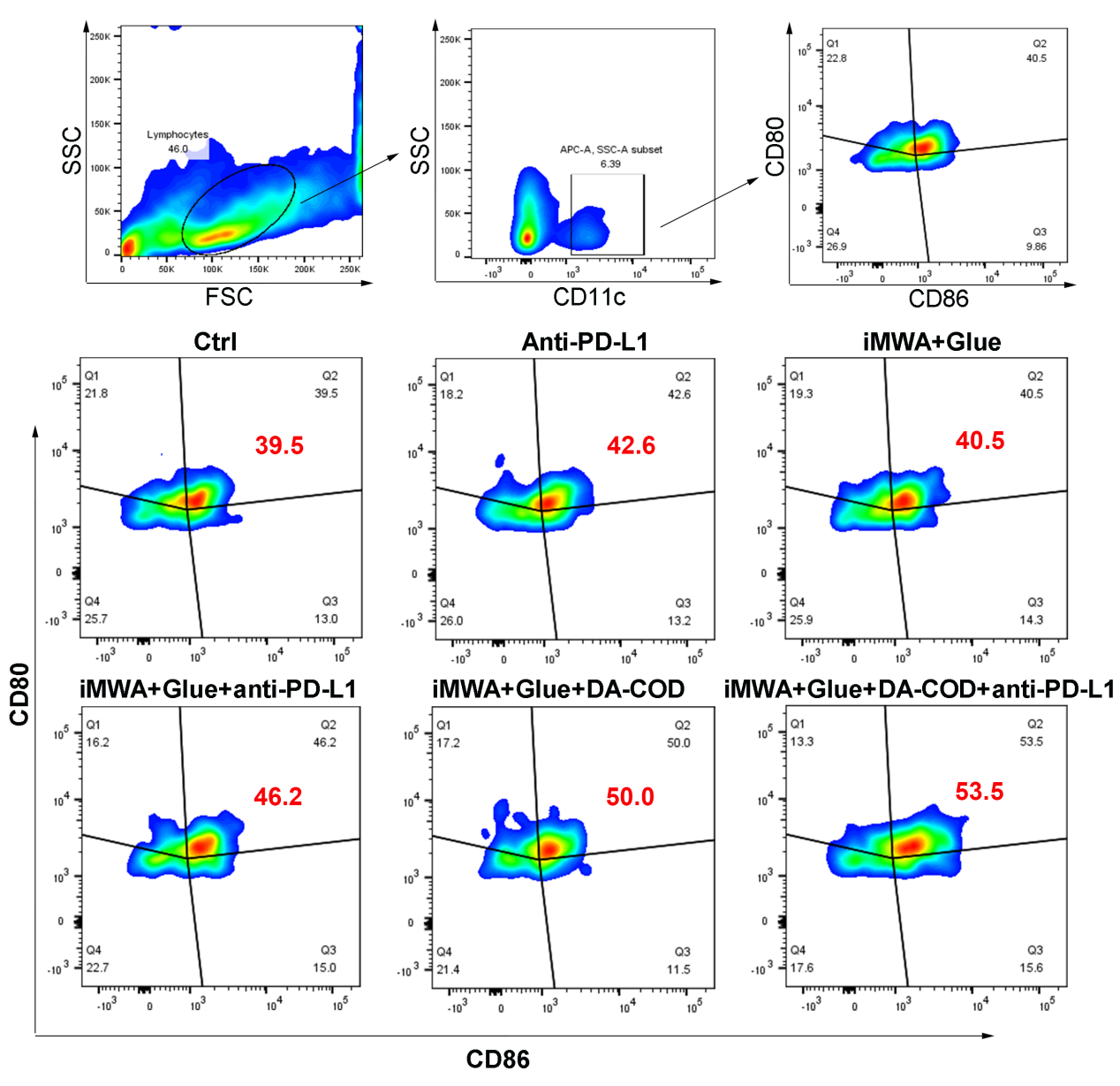


**Figure S9.** Flow cytometry showing the DC maturation status in the drain lymph nodes adjacent to the primary tumors after various treatments.

**
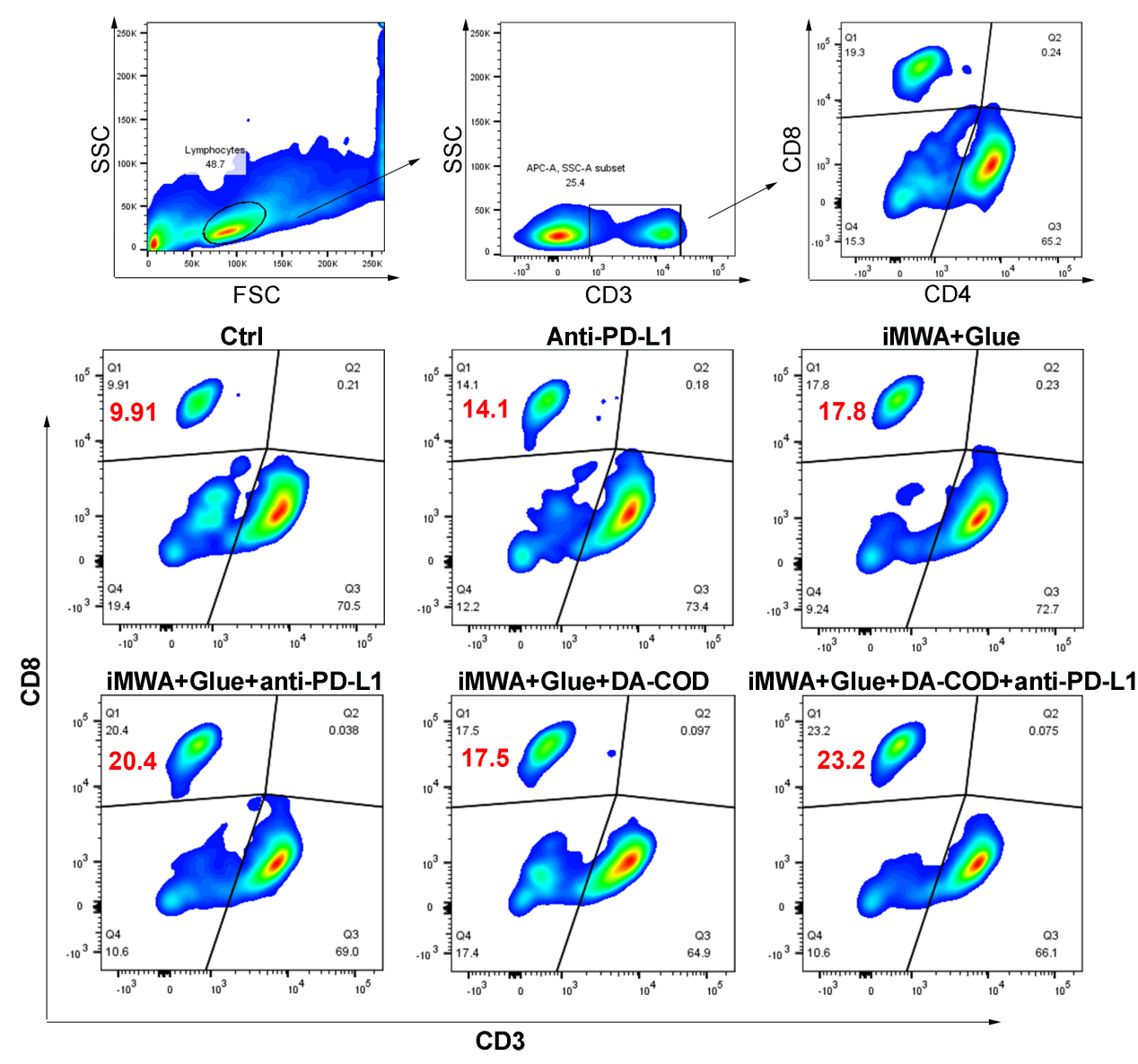
**

**Figure S10.** Flow cytometry showing the frequencies of CD3^+^ CD8^+^ T cells inside distant tumors after various treatments, as indicated. Glue, OD-HCS hydrogel.


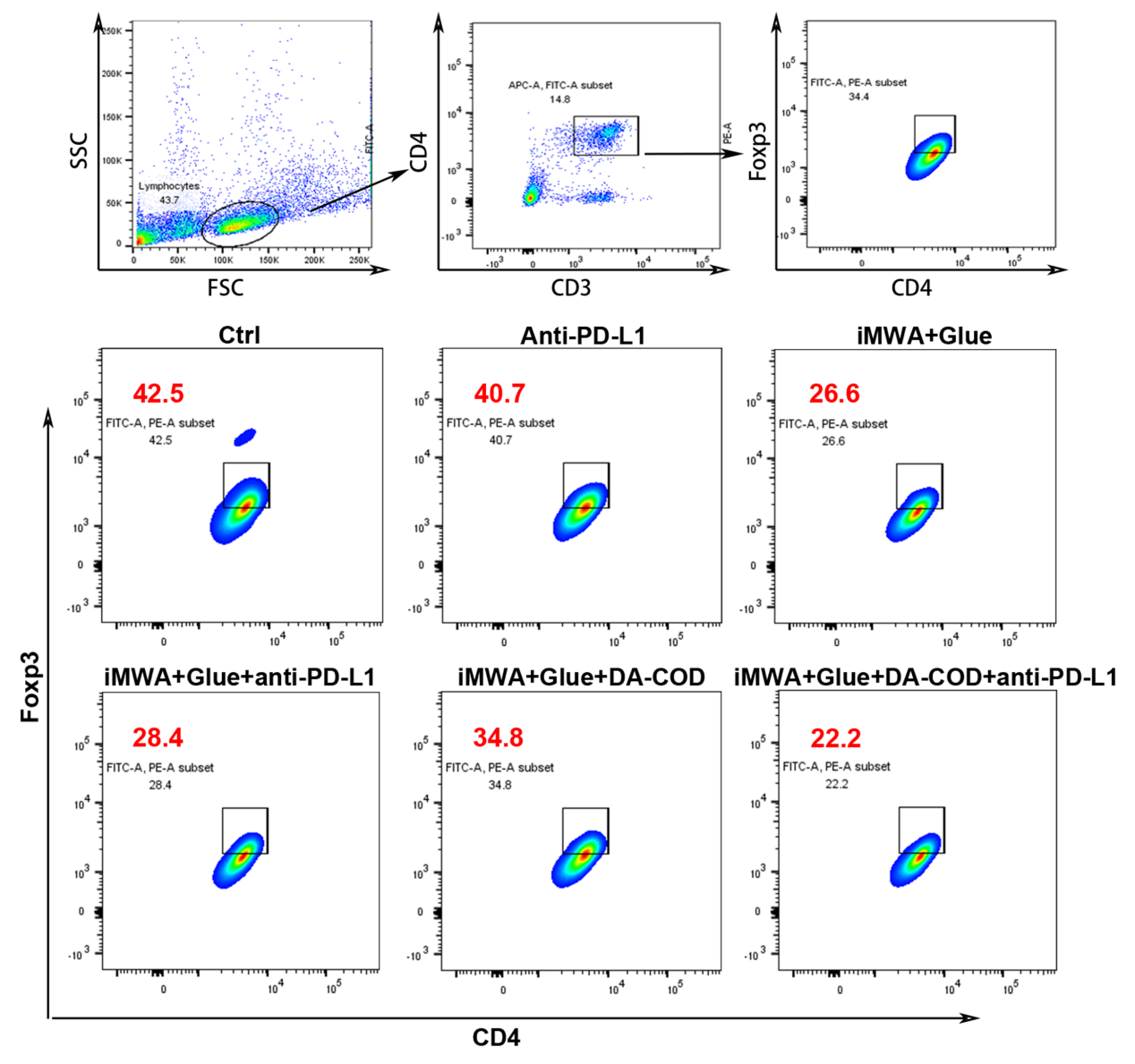


**Figure S11.** Flow cytometry showing the frequencies of CD3^+^ CD4^+^ Foxp3^+^ Tregs inside distant tumors after various treatments, as indicated. Glue, OD-HCS hydrogel.


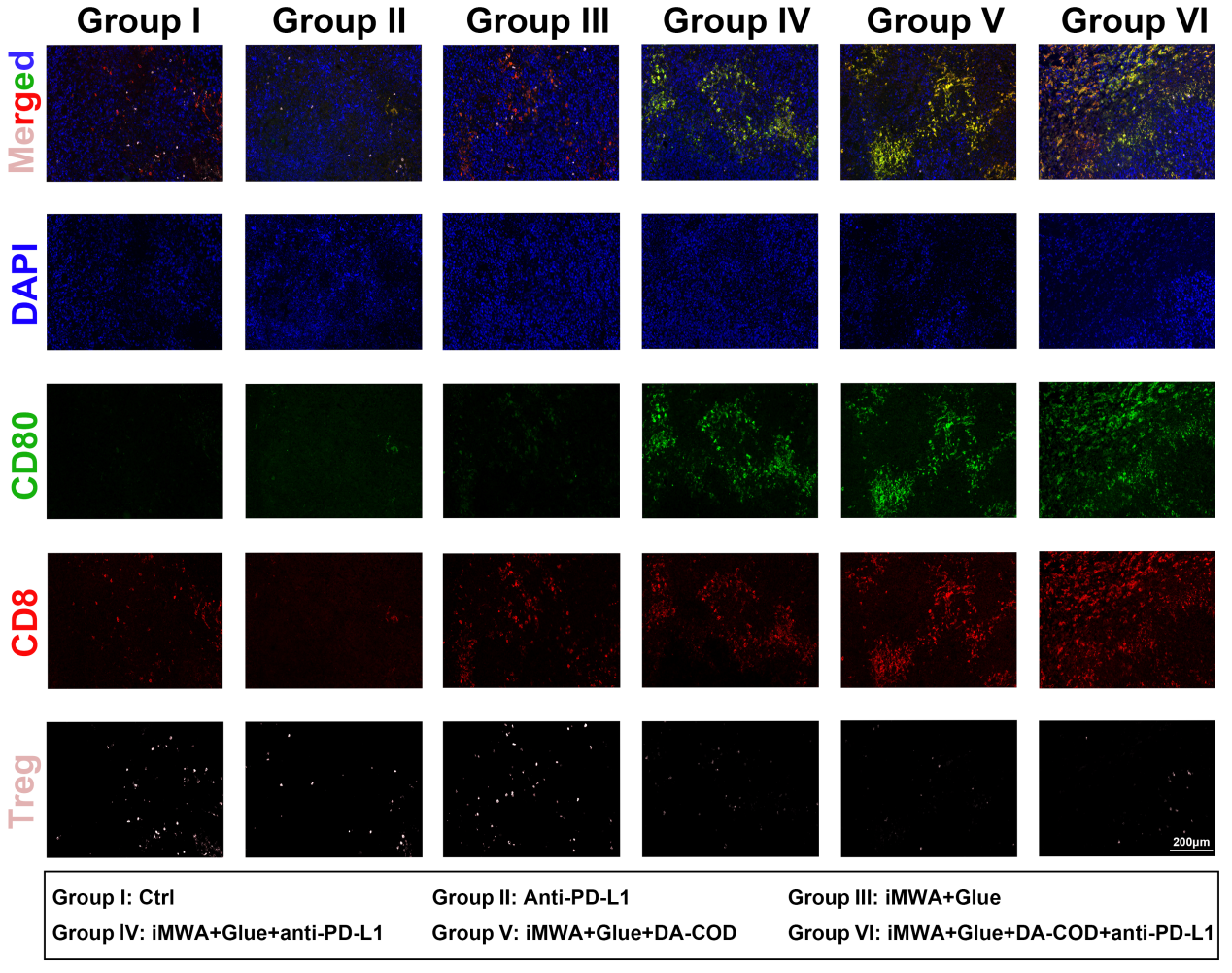


**Figure S12.** Confocal images of tumor slices collected from H22 tumor-bearing mice after different treatments, as indicated, and stained with DAPI (blue), CD80 (green), CD8 (red), and Tregs (pink).


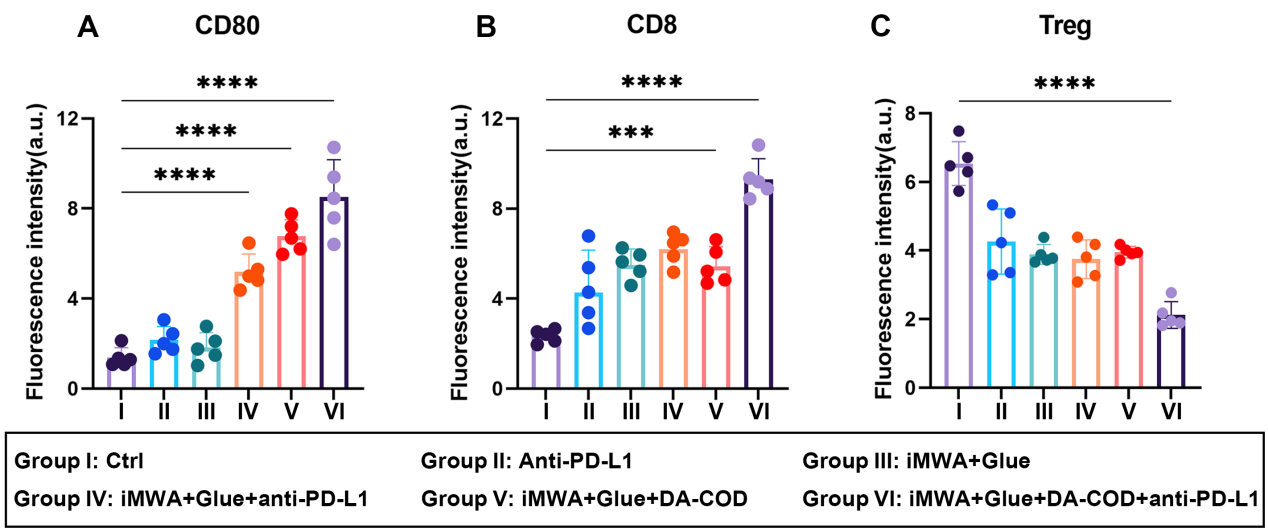


**Figure S13. (A-C)** Semiquantitative analysis of intratumoral CD80 (A), CD8 (B), and Treg (C) fluorescence signals. Glue, OD-HCS hydrogel. The data are presented as the means ± SEMs; n = 5 biologically independent samples. *p < 0.05, **p < 0.01, ***p < 0.001, ****p < 0.0001.


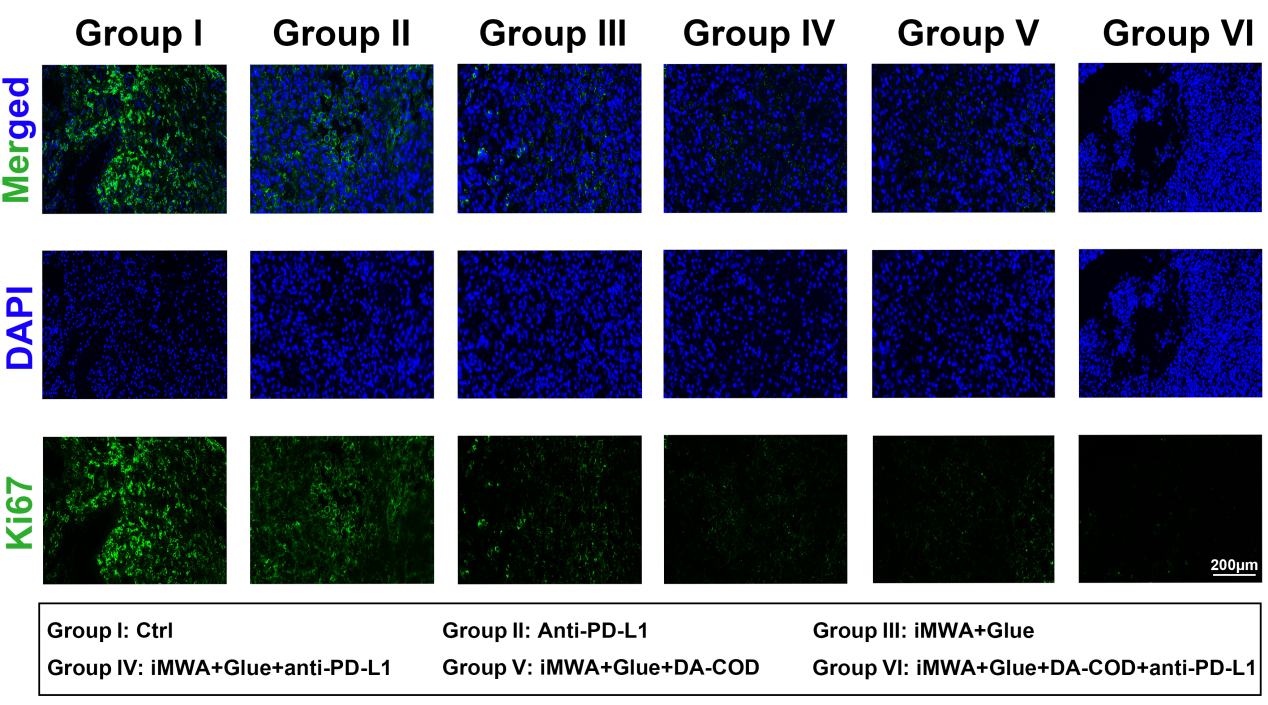


**Figure S14.** Confocal images of tumor slices collected from H22 tumor-bearing mice after different treatments, as indicated, and stained with DAPI (blue) and Ki67 (green).

**
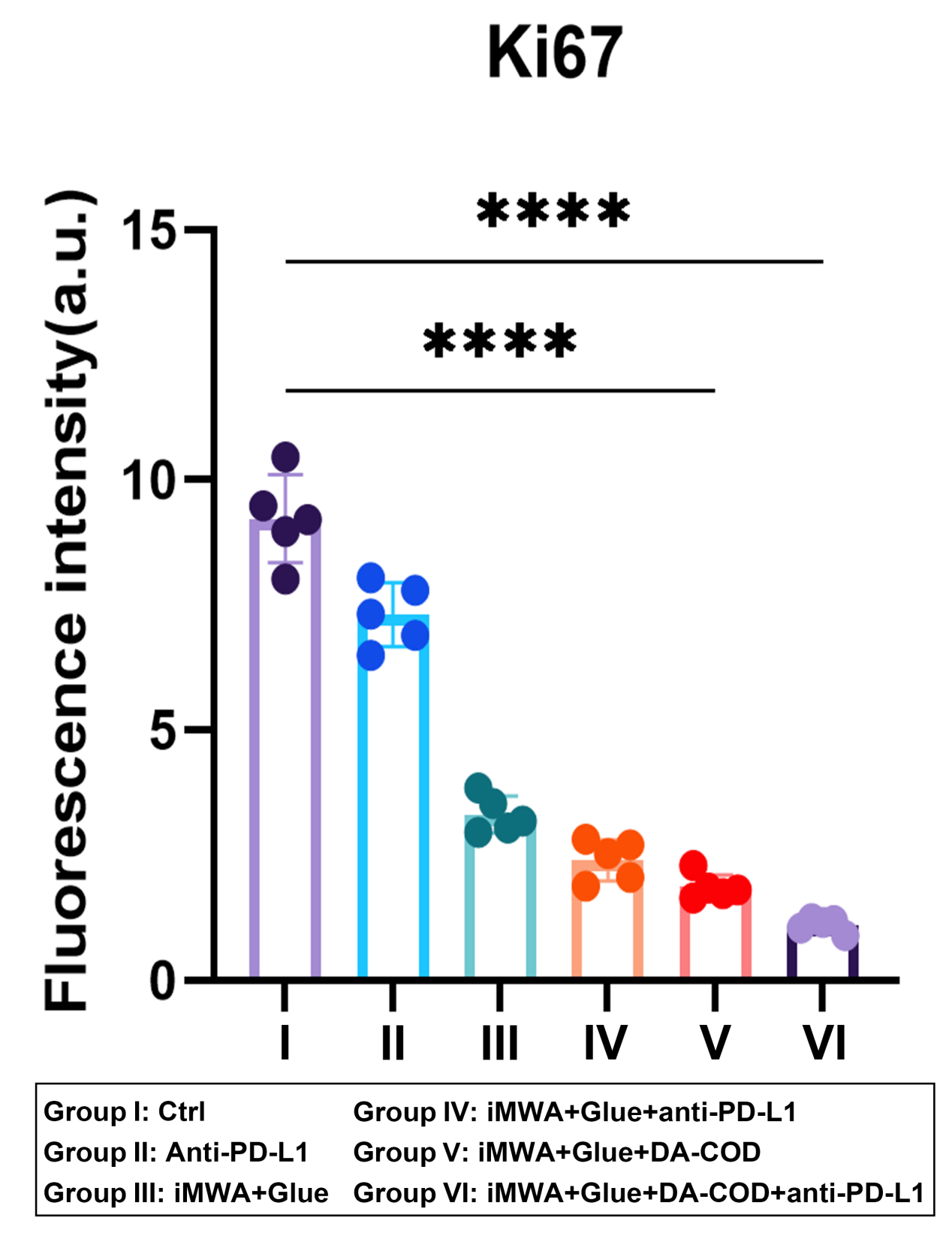
**

**Figure S15.** Semiquantitative analysis of intratumoral Ki67 fluorescence signals in Figure S14. Glue, OD-HCS hydrogel. The data are presented as the means ± SEMs; n = 5 biologically independent samples. *p < 0.05, **p < 0.01, ***p < 0.001, ****p < 0.0001.

**
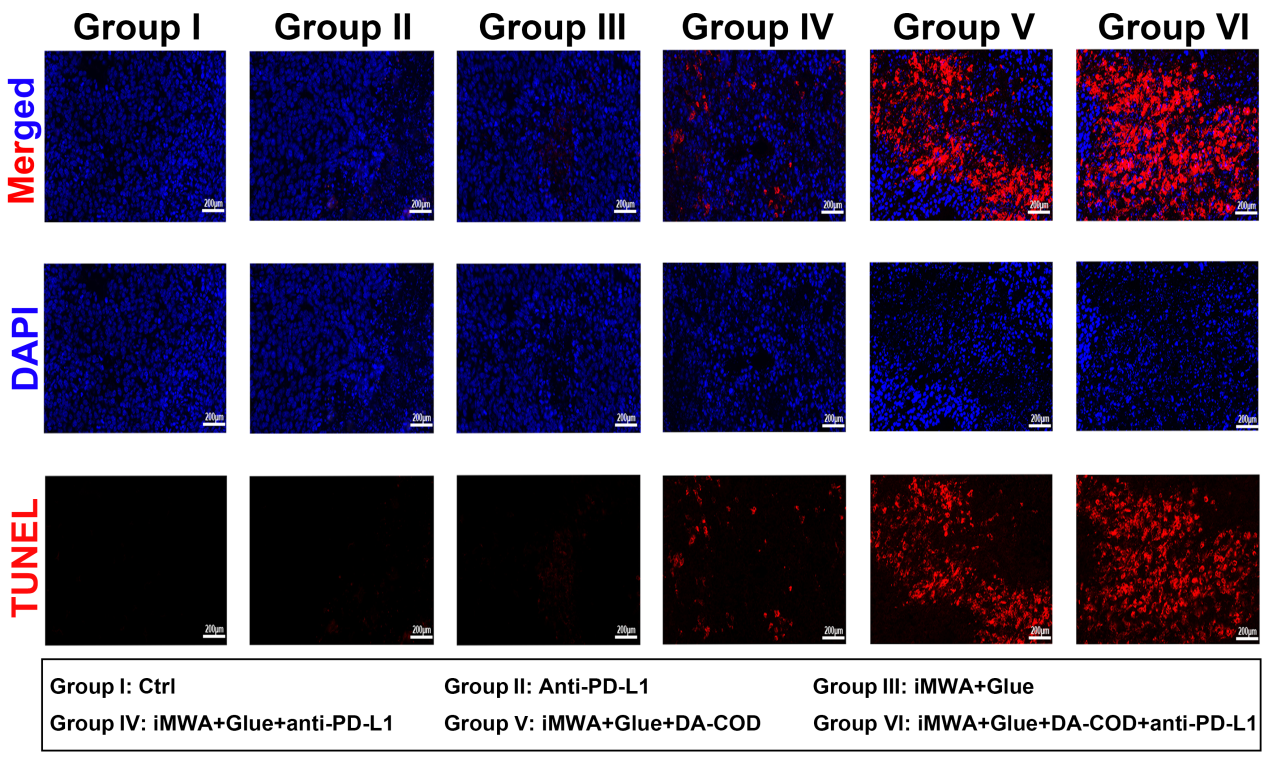
**

**Figure S16.** Confocal images of tumor slices collected from H22 tumor-bearing mice after different treatments, as indicated, and stained with DAPI (blue) and TUNEL (red).

**
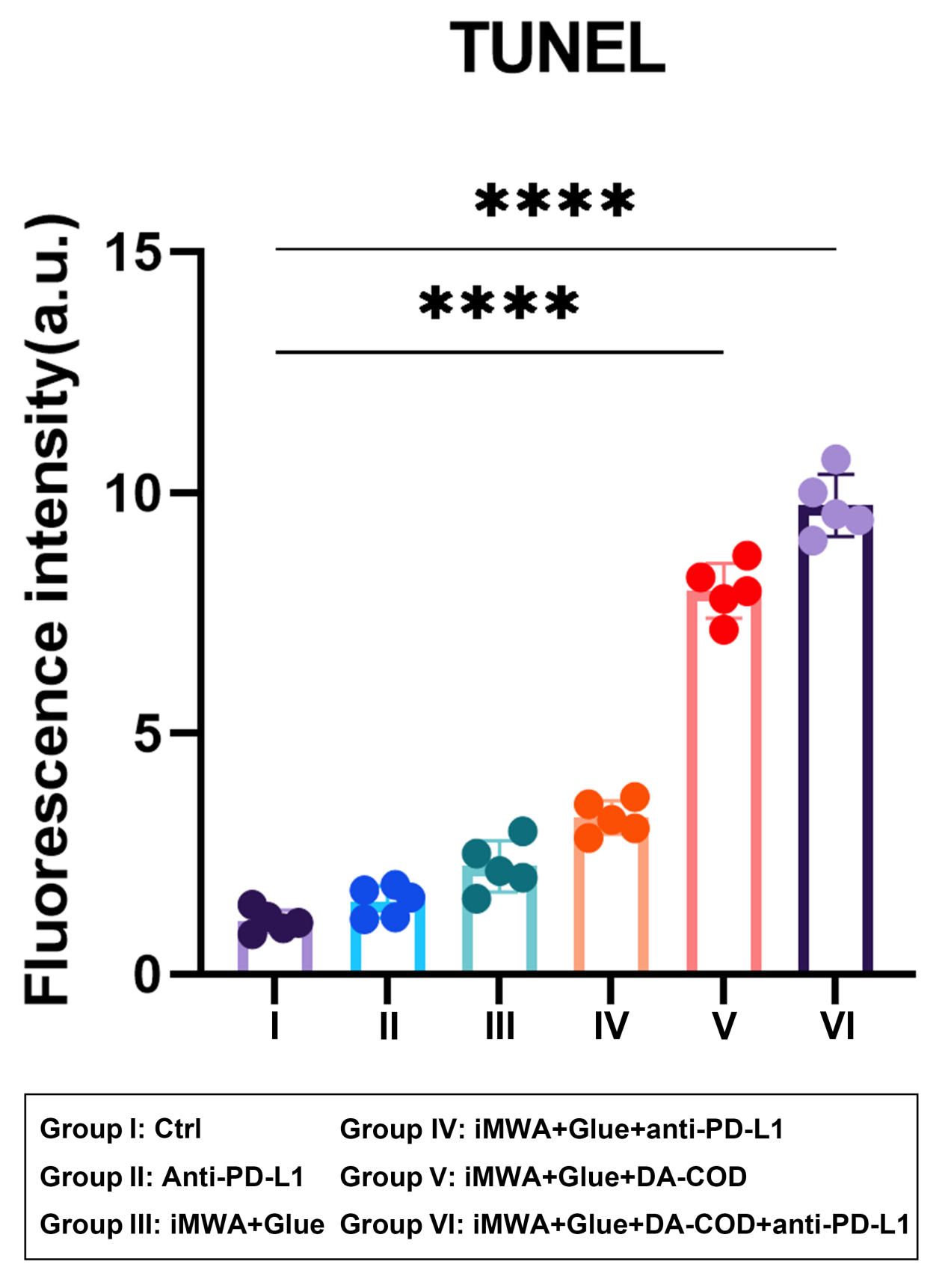
**

**Figure S17.** Semiquantitative analysis of the intratumoral TUNEL fluorescence signals in Figure S16. Glue, OD-HCS hydrogel. The data are presented as the means ± SEMs; n = 5 biologically independent samples. *p < 0.05, **p < 0.01, ***p < 0.001, ****p < 0.0001.


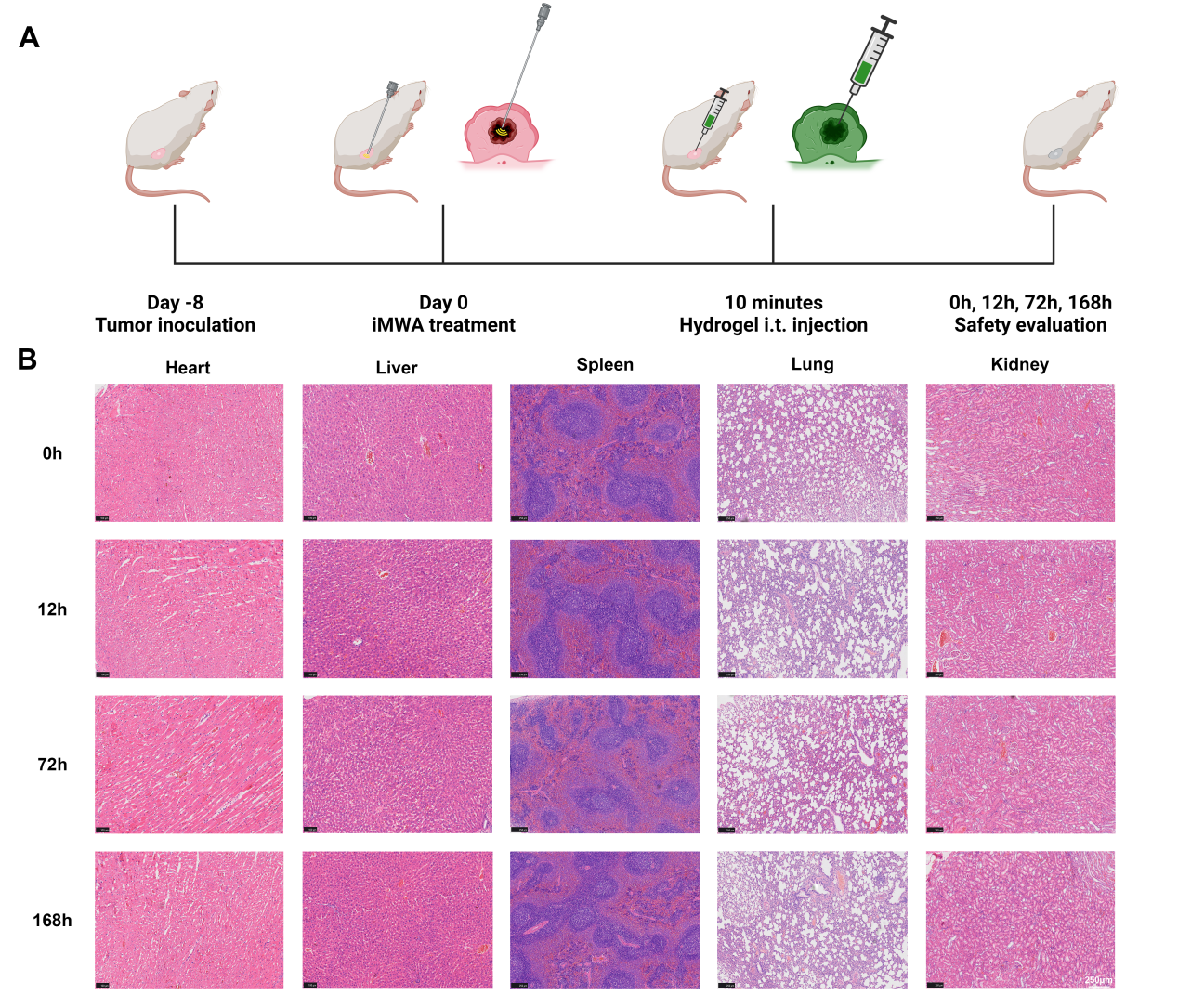


**Figure S18.** Evaluation of the safety of cholesterol-fueled catalytic hydrogel injection after MWA. **(A)** Schematic illustration of the experimental schedule used for the safety evaluation. **(B)** H&E staining of the main organ slices (heart, liver, spleen, lung, and kidney) collected from the mice at various time points (0, 12, 72, 168 h) after iMWA treatment and i.t. hydrogel injection.

**
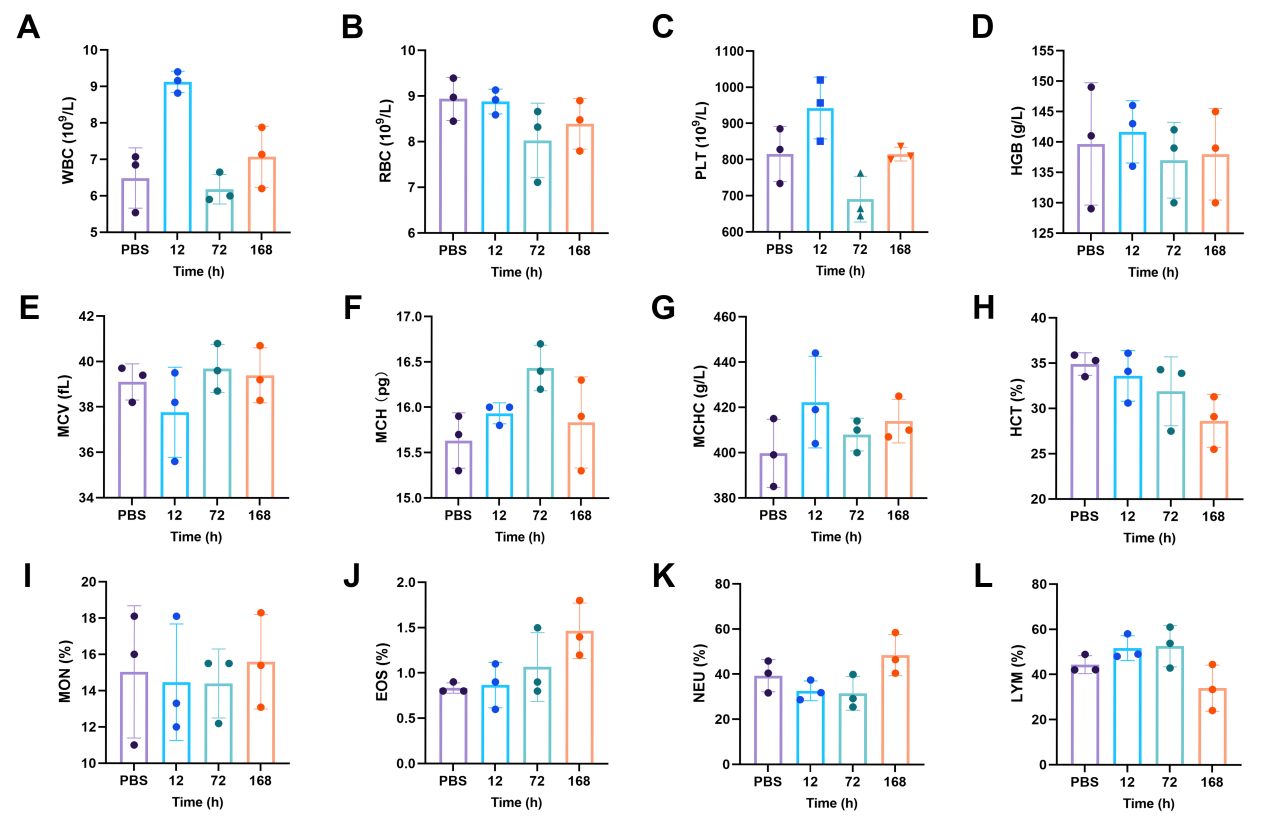
**

**Figure S19. (A-L)** The routine complete blood panel counter test and serum biochemistry analysis of healthy BALB/c mice evaluated at various time intervals post iMWA treatment and i.t. hydrogel injection. The measured parameters included white blood cells (WBCs, A), red blood cells (RBCs, B), platelets (PLTs, C), hemoglobin (HGB, D), mean corpuscular volume (MCV, E), mean corpuscular hemoglobin (MCH, F), mean corpuscular hemoglobin concentration (MCHC, G), hematocrit (HCT, H), percentage of monocytes (MON%, I), percentage of eosinophils (EOS%, J), percentage of neutral cells (NEU%, K) and percentage of lymphocytes (LYM%, L). The data are presented as the means ± SEMs; n = 3 biologically independent samples.
